# Nanoparticulate Photoluminescent Probes for Bioimaging: Small Molecules and Polymers

**DOI:** 10.3390/ijms23094949

**Published:** 2022-04-29

**Authors:** Sanghyuck Lee, Chul Soon Park, Hyeonseok Yoon

**Affiliations:** 1 Department of Polymer Engineering, Graduate School, Chonnam National University, 77 Yongbong-ro, Buk-gu, Gwangju 61186, Korea; sanghyuck89@gmail.com; 2 Drug Manufacturing Center, Daegu-Gyeongbuk Medical Innovation Foundation (DGMIF), Daegu 41061, Korea; cspark@kmedihub.re.kr; 3 School of Polymer Science and Engineering, Chonnam National University, 77 Yongbong-ro, Buk-gu, Gwangju 61186, Korea

**Keywords:** photoluminescence, bioimaging, polymer dots, near-infrared, quantum dots, small-molecule probes, in vivo

## Abstract

Recent interest in research on photoluminescent molecules due to their unique properties has played an important role in advancing the bioimaging field. In particular, small molecules and organic dots as probes have great potential for the achievement of bioimaging because of their desirable properties. In this review, we provide an introduction of probes consisting of fluorescent small molecules and polymers that emit light across the ultraviolet and near-infrared wavelength ranges, along with a brief summary of the most recent techniques for bioimaging. Since photoluminescence probes emitting light in different ranges have different goals and targets, their respective strategies also differ. Diverse and novel strategies using photoluminescence probes against targets have gradually been introduced in the related literature. Among recent papers (published within the last 5 years) on the topic, we here concentrate on the photophysical properties and strategies for the design of molecular probes, with key examples of in vivo photoluminescence research for practical applications. More in-depth studies on these probes will provide key insights into how to control the molecular structure and size/shape of organic probes for expanded bioimaging research and applications.

## 1. Introduction

Biological imaging technology is based on various strategies for analyzing and solving the mysteries of the human body, understanding the roots of disease, and achieving individualized or personalized medicine. Therefore, bioimaging requires probes with very high specificity and sensitivity. Accordingly, the exploration of materials with excellent photoluminescence properties and suitable strategies for their synthesis and application is needed to analyze the biological microenvironment and associated processes. Recent non-invasive photoluminescence imaging techniques appear to represent simple technology; however, these techniques are in fact highly versatile with real-time monitoring capabilities, providing unprecedented spatial and temporal resolution. Although numerous studies proposing novel imaging strategies have been published recently, further development of phosphors with higher brightness and luminous properties is necessary. In addition, the recent tremendous advancement of clinical technology and instruments for photoluminescence imaging induction surgery in patients has led to the need for further photoluminescence development, especially for the development of radioactive and biocompatible fluorophores [1,2,3]. 

Photoluminescence imaging technology has specific advantages for biomedical imaging applications, including high sensitivity, low cost, and high spatial/temporal resolution. Studies have been conducted on the use of various materials such as small-molecule luminophores [4,5,6], polymer dots (Pdots) [7,8,9], single-walled carbon nanotubes [10,11,12,13,14], semiconductor quantum dots (QDs) [15,16,17,18,19,20,21,22,23], and rare Earth-doped nanoparticles [24,25,26] as fluorescent probes. However, each of these probes has limitations such as a low quantum yield, low solubility, high cytotoxicity, and low photostability. One of the main areas of focus in the field of fluorescence-based bioimaging applications is far-infrared and near-infrared (NIR) fluorescence (650–1000 nm). Fluorescence emitted from the NIR range has recently been used in clinical imaging-guided cancer surgery, demonstrating excellent potential for improvement in results [27,28,29,30]. However, the limited selection of available photoluminescence probes is an important obstacle in applying photoluminescence imaging to a wide range of biological samples. Most biological species such as water, lipids, melanin, oxyhemoglobin, and deoxyhemoglobin absorb very little light and exhibit low scattering within the NIR region [31]. In addition, since biological tissues have very low autofluorescence within the NIR region, the use of photoluminescence probes emitting in this region may result in high signal-to-noise contrast. More importantly, visible light can penetrate only a few micrometers within the tissue, whereas NIR rays (700–900 nm) can penetrate up to a few millimeters through the blood or tissue, providing a clear surgical guide with minimal tissue damage [32,33]. 

Despite these achievements, more advanced bioimaging techniques are needed to further increase the properties of absorption, scattering, and autofluorescence transmittance in biological tissues. Recently, infrared spectral windows were classified into NIR-I (700–1000 nm) and NIR-II (1000–1700 nm) [31,34]. Images using photoluminescence probes emitting in higher-wavelength bands can reduce autofluorescence and photon attenuation and obtain a higher signal-to-background ratio (SBR); however, the low quantum yield and low biocompatibility remain important obstacles to their clinical application [35,36,37]. In addition, although many inorganic and carbon nanomaterials (e.g., single-walled carbon nanotubes, QDs, and rare Earth-doped nanoparticles) have been developed as NIR-II probes, their immunoadsorption, biocompatibility, and elimination after use remain important issues to be improved. Continuous development and potential application of NIR probes in the real-time imaging of biological processes and analytes in vivo will provide mechanistic insights into the underlying biology and disease pathology, leading to clinical translation to ultimately realize overall improvements in disease diagnosis and treatment patterns. 

In this review, we introduce recent studies on synthesized small molecules and semiconducting polymer-based Pdots as photoluminescent probes for bioimaging. We compare the advantages and disadvantages of these small-molecule and polymeric probes according to their characteristics with respect to their application potential. We also provide a brief overview of studies using carbon dots (CDs), graphene quantum dots (GQDs), metal-organic frameworks (MOFs), and covalent organic frameworks (COFs). We hope that this review will help researchers select the most suitable bioimaging probe material for future studies, and we further discuss some challenges and exciting opportunities in this emerging field.

## 2. Overview of Several Photoluminescent Probes

### 2.1. Small-Molecule Probes

**Figure 1:**
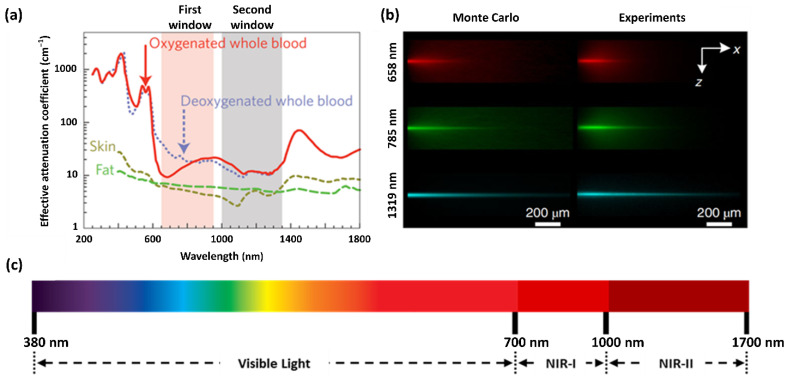
(**a**) Two optical windows showing the high transparency in biological tissues and fluids. Reprinted with permission from Ref. [31]. Copyright 2009, Springer Nature. (**b**) Photographs of experimental results in tissue penetration. Reprinted with permission from Ref. [43]. Copyright 2019, Springer Nature. (**c**) Spectral ranges for visible light, near-infrared (NIR)-I, and NIR-II showing the corresponding wavelengths.

Optical imaging based on small-molecule photoluminescence probes has attracted substantial attention owing to its characteristic advantages. In particular, these probes can help to visualize non-invasive and diverse biological components, enabling the real-time monitoring of an organ’s dynamic processes at the molecular, cellular, and living organism levels. Above all, optical imaging using small-molecule photoluminescence probes is a fast, sensitive, specific, and multiplexed method to detect analytes [38,39,40,41,42]. However, optical imaging is generally constrained by the electromagnetic spectrum region used for biological sampling. In the optical window shown in Figure 1a, the light absorption/emission ranges of the skin/fat and oxidized/deoxygenated whole blood are particularly low at 650–950 nm and 1000–1350 nm, referred to as the first and second windows, respectively. As relatively high light transmittance is guaranteed in these windows, photoluminescence probes in the NIR region can provide deeper penetration and higher image quality than photoluminescence probes emitting in the visible-light region [31,43]. To accurately detect and monitor significant biomolecules and molecular events in real time, numerous reasonably designed NIR-I fluorescent probes have recently been developed, which have also been used to aid surgical imaging treatments such as the removal of tumor tissue and photothermal therapy. Optical wavelengths in regions above 1000 nm further minimize the number of photons absorbed or scattered by biomolecules and media, providing deeper penetration and good spatial resolution with lower non-target auto-fluorescence. 

**Figure 2:**
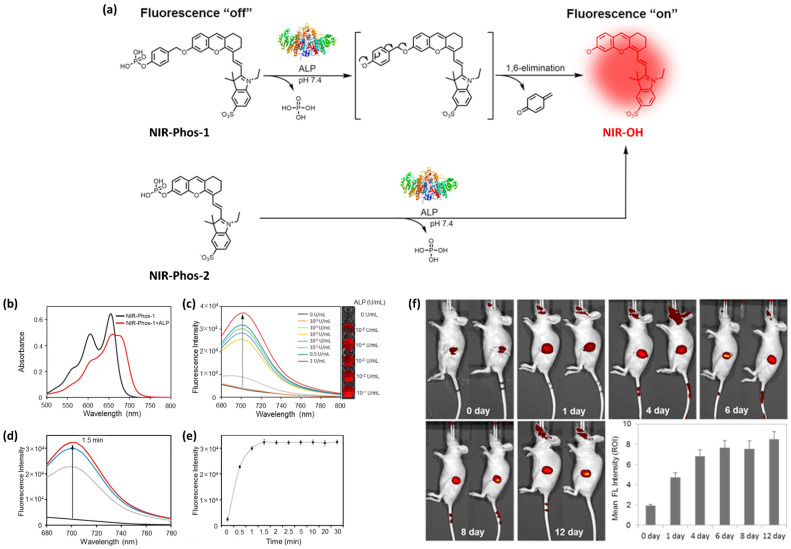
(**a**) Schematic illustration of the fluorescence “turn-on” process of near-infrared (NIR) probes triggered by alkaline phosphatase (ALP). (**b**) Ultraviolet-visible absorption of NIR probes (5 μM) and (**c**) fluorescence emission spectra and fluorescence images (excitation = 685 nm) of NIR probes (5 μM) with different concentrations of ALP (0–10^−5^ U mL^−1^; the reaction time for all concentrations was 1.5 min) in Tris-HCl buffer (10 mM, pH 7.4). (**d**) Fluorescence emission spectra of NIR-Phos-1 (5 μM). (**e**) Fluorescence emission spectra of NIR-Phos-1 (5 μM) with ALP (0.1 U mL^−1^) over different time periods (0–30 min) at room temperature. The spectra were obtained at 0.5 min intervals. (**f**) Fluorescence images of NIR-Phos-1-labeled CDHA scaffolds implanted in mice. Reprinted with permission from Ref. [44]. Copyright 2018, Elsevier.

Several valuable strategies have been used for manufacturing meaningful bioimaging devices. As a typical bioimaging study, in 2018, our group [44] also highlighted the importance of optically detecting alkaline phosphatase (ALP) with NIR small-molecule probes (Figure 2). As schematically illustrated in Figure 2a, two different NIR fluorescent probes were synthesized, consisting of a phenolic dihydroxanthene fluorophore core and a phosphate with a sulfonate group to improve water solubility. Although these NIR probes are almost completely nonfluorescent, they emit fluorescence upon ALP-dependent catalytic dephosphorylation, leading to a concentration- and time-dependent “turn-on” fluorescence mechanism (Figure 2b). The ALP level in the blood can be used to diagnose several conditions, including bone disorders and liver dysfunction. As displayed in Figure 2c, in vivo monitoring of ALP signal changes was successfully achieved with an NIR probe-labeled calcium-deficient hydroxyapatite (CHDA) scaffold implanted into mice, indicating that the NIR probe can serve as an indicator of osteoblast activity during early osteoblast differentiation.

Recent studies have achieved clearer photoluminescence imaging with an improved SBR in the NIR-II region (1000–1700 nm) than in the NIR-I region because light scattering is reduced further in the former than in the latter [45,46,47,48]. In this vein, the focus of recent related studies has been to develop new fluorescent probes that absorb or emit in the NIR-II region for optical imaging. Small molecule-based fluorescent probes have the well-known advantages of a core structure and the ability for specific modifications to target a biomarker according to their physical and optical properties. Therefore, it is easy to design a specific structure and to establish the optimal strategy for bioimaging with NIR-II fluorescent reagents. Consequently, the development of small molecule-based organic fluorophores for the NIR-II region is a major research hotspot in the field of biological imaging and for the exploration of associated chemical approaches.

### 2.2. Pdots

Recently, Pdots, which have semiconductor characteristics, have emerged as powerful probes in various applications such as bioimaging/biosensing and photodynamic therapy [8,49,50]. Compared with conventional photoluminescence probes, rationally designed Pdots may exhibit high single-particle brightness, a high radiative rate, and outstanding photostability. The fluorescence of Pdots is derived from the exciton transition from the singlet state to the ground state, and phosphorescence is accomplished by the exciton transition from the triplet state to the ground state. By contrast, thermally activated delayed fluorescence (TADF) is a distinct mechanism in which the exciton transition from the triplet excited state to the singlet excited state occurs through a thermally activated reverse inter-system crossing process. TADF consists of both short (nanosecond scale) and long (micro- to millisecond scale) emission lifetime regimes, facilitating its use in time-resolved fluorescence imaging [51,52,53,54,55]. Because TADF takes advantage of the long-lived triplet excited states that are more vulnerable to luminescence quenching, the TADF process should be protected from undesirable quenching mechanisms, making it more useful for biological imaging in practice. One method to protect against quenching is to encapsulate TADF fluorophores within water-soluble nanoparticles; however, over time, the fluorophores are released from the nanoparticles, thereby reducing the fluorescence signal’s intensity. A typical solution to overcome this limitation is to use a semiconducting polymer with an intrinsic TADF property, such as hydrophilic polymers, for producing a nanoparticulate polymer probe. Similarly, other mechanisms of action such as Förster resonance energy transfer, photoinduced electron transfer, aggregation and disaggregation-induced emission, intramolecular charge transfer (ICT), and motion-induced changes in emission can be more efficiently realized within polymer matrices. Furthermore, the surface of Pdots can be modified to enhance their suitability for bioimaging. For example, Pdots generally consist of hydrophobic π-conjugated polymers. However, when mixed with a hydrophilic polymer such as polyethylene glycol (PEG), Pdots can minimize non-specific binding with non-target species. This versatile surface functionalization ability of Pdots further provides various opportunities for their applications, such as in sensors, cell labeling, and bioimaging. Moreover, the appropriate design and strategy for the surface functionalization of Pdots can facilitate binding to specific molecules covalently when targeting tumor cells and stem cells. Recently, sensing platforms have been designed for detecting chemical ions and for molecular recognition in point-of-care diagnostic devices based on Pdots, showing high sensitivity and accuracy [56,57,58]. Due to their characteristics of improved luminosity and high luminescence brightness, Pdots can also be used in selective cell targeting to provide a reverse tile platform for theragnostics. Therefore, many recent studies have utilized Pdots to discover applications for intracellular single-particle tracking, lateral flow or blot-style analysis, and immuno-labeling of cancer cells [59,60,61,62,63,64,65].

### 2.3. CDs and GQDs 

Due to their brightness and photostability properties, inorganic semiconductor QDs have attracted substantial attention as a potential alternative to organic fluorescent probes in bioimaging. However, their drawbacks, including poor water solubility, blinking, and intrinsic toxicity, pose challenges for the use of QDs in bioimaging applications [66,67,68,69], leading to further research attempting to enhance their properties. 

Since the discovery of luminescent CDs in 2004 [70], extremely diverse investigations have been conducted on synthetic methods, the exploration of reaction mechanisms, and their potential applications due to their remarkable characteristics such as tunable optical properties, good photostability, and superb biocompatibility [71,72,73,74,75]. In particular, CDs not only have surface functional groups enabling post-synthetic functionalization but also have high biocompatibility, offering great potential in applications such as the real-time tracking of drugs, tumor therapy, and bioimaging/biosensing. Furthermore, the methods of preparing CDs are mostly simple, cost-effective, and environmentally friendly. CDs can be prepared in one-step procedures, using microwave and hydrothermal methods, and with almost any type of materials (e.g., amino acids, glucose, dopamine, and citric acid) as precursors, including those obtained from natural sources. Recently, diverse, effective methods for manufacturing photoluminescent CDs have been developed, which can be divided into top-down and bottom-up approaches [76,77,78,79]. Laser ablation, chemical oxidation, electrochemical oxidation, and arc discharge methods are examples of top-down methods, whereas microwave irradiation, thermal decomposition, hydrothermal treatment, and plasma treatment are examples of bottom-up methods. By researching further developments of these various methods, the applications of CDs can be expanded, with remarkable results expected in the future.

GQDs consist of nanometer-sized fragments of graphene layers with chemical functional groups. Graphene has a hexagonal lattice and consists of two-dimensional sheets [80]. Furthermore, the highly ordered and closely carbon atom-packed structure grants graphene unique and useful properties [81,82]. One such property is the zero-energy bandgap due to the overlapping of graphene’s valence and conduction bands. However, the zero bandgap is attributed only to immaculate graphene, which refers to an infinite-dimension graphene with no defects. By contrast, actual graphene contains defects with a finite value in physical dimensions, resulting in a non-zero bandgap. Consequently, GQDs consisting of nanoscale fragments of graphene exhibit quantum confinement effects with a tunable bandgap [83,84,85,86]. Diverse research has revealed that photoluminescence excitation and emission wavelengths can be changed by adjusting the size of GQDs, changing the surface properties or introducing dopants such as boron and nitrogen atoms into the carbon lattice of graphene. The photoluminescence characteristics of GQDs can be adjusted to improve their suitability for optical applications and effectiveness in bioimaging/biosensing. In addition to a dopant within the lattice, hydroxyl and carboxyl groups, which are oxygen-rich functional groups, exist on the edge of GQDs. These functional groups enhance the water solubility, thereby facilitating the biological applications of GQDs.

### 2.4. MOFs and COFs

MOFs consist of organic ligands and metal ions (or clusters). Since MOFs have remarkable advantages such as a large surface area, high porosity, and tunable chemical composition, they have shown strong potential for various sensing, catalytic, and biological applications [87,88,89]. In particular, many recent studies demonstrated the use of MOFs with photoluminescence properties in bioimaging/biosensing and disease treatment [90]. The organic ligands of MOFs directly influence their luminescent characteristics. In addition, for biological applications, the organic ligands of MOFs have to be carefully selected by considering the biocompatibility and toxicity. Furthermore, the fluorescence range of MOFs can be expanded by combination with other luminescent species. 

COFs have been continuously studied since their discovery in 2005 [91]. COFs feature organic porous networks that are covalently linked with organic materials. In contrast to MOFs, COFs consist of light elements such as hydrogen, boron, carbon, nitrogen, and oxygen. Furthermore, in contrast to amorphous organic polymers, COFs have a well-defined two- or three-dimensional crystalline structure. COFs have attracted substantial research attention due to their beneficial characteristics such as a tunable chemical structure/functionality, porosity, high crystallinity, and large surface area. Consequently, COFs show strong potential for applications in optoelectronics, such as in catalysts, energy storage/conversion devices, and in sensors capable of detecting explosives, ammonia, chiral species, small molecules, and DNA [92,93,94,95,96,97,98,99,100,101,102]. In general, the π-conjugated skeleton endows COFs with a unique fluorescence property. Most studies on COFs have mainly focused on constructing conjugated structures using different chemical units or establishing different types of bonds between them.

Table 1 summarizes the advantages and disadvantages of the above-mentioned photoluminescent probes. The following sections address the characteristics of the small-molecule and polymeric probes for bioimaging applications, with remarkable recent research examples.

## 3. Small-Molecule Probes for Bioimaging

**Figure 3:**
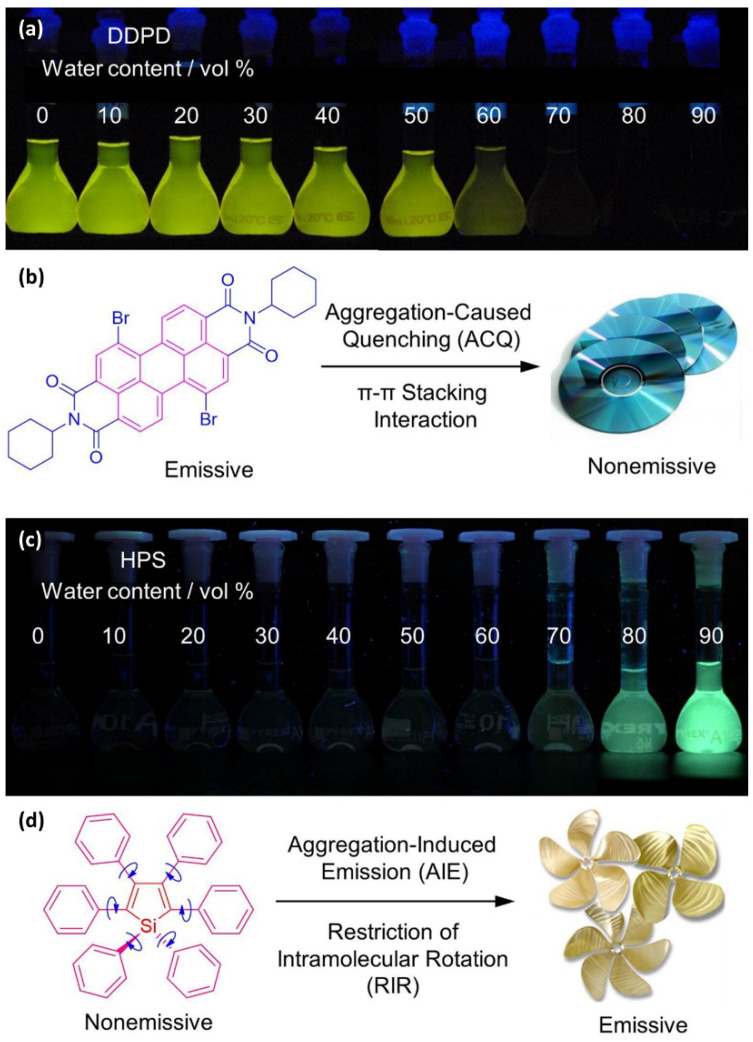
Fluorescent digital photographs of (**a**) fluorescein and (**c**) hexaphenylsilole (HPS) solutions with different bad solvent fractions. (**b**) Fluorescein molecules are disk-shaped and become non-emissive when forming aggregates where a strong π−π interaction quenches the emission. (**d**) HPS molecules are propeller-shaped and become highly emissive when forming aggregates where the intramolecular rotation is restricted. Reprinted with permission from Ref. [155]. Copyright 2018, American Chemical Society.

Small molecule-based organic fluorescent probes have a well-defined structure and can be designed to be tunable for various applications according to their spectral properties based on their optical and physical characteristics. Because of these advantages, small molecule-based organic fluorescent probes have been extensively studied for bioimaging. There are two notable mechanisms that underly the luminescence processes at the molecular level [155]. Mostly, small-molecule probes show concentration-dependent luminescence behavior. Luminescence from the small molecules is weakened or quenched at high concentrations. The quenching process can be associated with the formation of aggregates, a phenomenon that has been referred to as aggregation-caused quenching (ACQ). Figure 3a presents a typical example of the ACQ effect. A diluted solution of *N*,*N*-dicyclohexyl-1,7-dibromo-3,4,9,10-perylenetetracarboxylic diimide (DDPD) in tetrahydrofuran (THF) is highly luminescent. However, its emission is weakened when water is added to THF. Since ACQ is inconvenient for practical applications, numerous efforts have been devoted to circumvent these limitations using chemical/physical methods and/or engineering processes [156,157,158,159,160]. Currently, the design of new molecular systems with aggregation-induced emission (AIE) is an active area of research on organic luminescent materials. Since the AIE effect is opposite to the undesirable ACQ effect, it is important to actively utilize the aggregation process instead of passively working against it. Figure 3b shows an example of the AIE effect. When hexaphenylsilole is dissolved in a good solvent such as THF, it is non-luminescent. However, intensive emission occurs after water is added to THF. Recently, there have been many efforts to develop strategies for converting ACQ to AIE. The Tang group reported that the restriction of intramolecular motions such as rotations and vibrations is considered to be the main factor in overcoming the ACQ phenomenon [161]. Various AIE systems have been explored with decorated aromatic rings, flexible chains, and intramolecular rings, such as coumarin, anthracene, naphthalene diimide, pyrrole, pyridine, diketopyrrolopyrrole (DPP), porphyrin, and their derivatives [162,163,164,165,166,167,168,169,170].

Likewise, understanding the role of the constituent chemical units in luminescent probes is of vital importance. Table 2 lists representative key chemical units that constitute the luminescent probes used for bioimaging. All these chemical units can play roles as electron donors, acceptors, or bridges connecting the donor/acceptor. First, tetraphenylethylene (TPE), an AIE-active fluorescent probe, has been used in diverse studies owing to its self-organization capability and ability to be incorporated into huge multi-component assemblies with ACQ fluorescent probes. TPE can be functionalized with guest organic species by electron conjugation. As a consequence, the absorption and emission properties of the structures change, making them highly usable in various applications. For example, anthracene, pyrene, and carbazole have been combined with TPE, and these functionalized forms of TPE exhibit distinct properties [171,172]. TPE has also been combined with triphenylamine (TPA) to adjust the emission wavelength [173]. In general, TPA is employed to achieve improved hole mobility. Benzobisthiadiazole (BBTD) has been used to construct various NIR fluorescent probes owing to its excellent photo-/thermostability and large Stokes shifts [174]. DPP has lactam rings that confer remarkable light-harvesting properties [175]. In particular, DPP-based luminescent compounds or materials can deliver outstanding photovoltaic performance in solar cells [176]. DPP with an inherently low bandgap can be chemically modified or coupled with other materials covalently or noncovalently (intermolecular π–π interaction), which offers possibilities to tune luminescence characteristics (e.g., lowering the bandgap for absorbing and emitting in the NIR region). Indocyanine green (ICG) is a well-known nontoxic NIR dye, exhibiting absorption at 785 nm and emission at 800 nm. ICG was approved by the Food and Drug Administration (FDA) in 1959 for evaluating hepatic function. Therefore, ICG is now widely used clinically in medical diagnostics, photoacoustic imaging, photodynamic therapy, and photothermal therapy. Thiophene consists of a five-membered heteroaromatic compound containing a sulfur atom at one position. Although thiophene is insoluble in water, it is soluble in many organic solvents. Because of the sulfur group, thiophene is delocalized in the π-electron system. In addition, the abundant electrons of thiophene can be used in various applications involving organic synthesis. Many theoretical and experimental studies, including analyses of the inhibition efficiencies of thiophene and its derivatives, have been performed to date. As a representative thiophene derivative, indeno[1,2-b]thiophene (IDT) is a powerful electron donor, which has a broad absorption wavelength range as well as outstanding charge-transfer properties. This molecule not only possesses an extended π-electron conjugation with excellent electron-donating capability due to its indole nitrogen atom but can further be synthesized with a one-step process using commercially available materials. In addition, IDT contains a symmetry-breaking unit, which contributes to the photovoltaic performance of its derivatives. Another thiophene derivative, 3,4-ethylenedioxythiophene (EDOT), acts as an electron donor and is typically utilized after polymerizing with poly(3,4-ethylenedioxythiophene) (PEDOT). Five-membered heterocycles, such as thiophene and pyrrole, and their derivatives have been used as the building blocks to prepare conducting polymers [177,178,179,180,181,182,183,184,185,186,187,188,189,190,191]. Among the existing conducting polymers, PEDOT shows particularly remarkable properties such as high and stable conductivity, excellent transparency, and high water solubility (with polystyrene sulfonate). Therefore, PEDOT has been widely employed in various research fields, ranging from a simple conductive coating to sensors, organic field-effect transistors, organic light-emitting diodes, and photovoltaics [177,178,179,180,181,182,183,192]. Furthermore, many different types of nitrobenzoxadiazole (NBD) fluorophore-based probes have been designed for various sensing and biological applications, in which NBD is most frequently used to facilitate the ICT transition in the molecular system. The cyclopropenium cation is the classic example of a Hückel 2π-electron aromatic ring system, which is highly stable due to its inherent aromaticity despite the apparent ring strain. Notably, the high degree of stability can be further enhanced through the incorporation of amino substituents onto the cyclopropenium ring. Moreover, the photophysical properties of the inherently fluorescent cyclopropenium cations can be tuned with those of other π-conjugated groups (e.g., naphthalene) to increase the quantum yield, molar attenuation coefficient, Stokes shift, and red-shifting absorbance and emission wavelengths. Lastly, triphenylphosphine (TPP)-based luminescent compounds have rarely been reported to date due to the low photoluminescence quantum yield. Recently, Zhang and colleagues [193] demonstrated that introducing steric hindrance groups to the TPP moiety and separating the orbitals involved in the transition can significantly suppress the non-radiative decay induced by the structural distortion of TPP in the excited state and could further increase the quantum yield by up to 0.89.

Importantly, these unit structures described in Table 2 are employed as building blocks to construct various molecular probes with ultraviolet (UV)-/NIR-range emissions for different purposes and applications.

**Figure 4:**
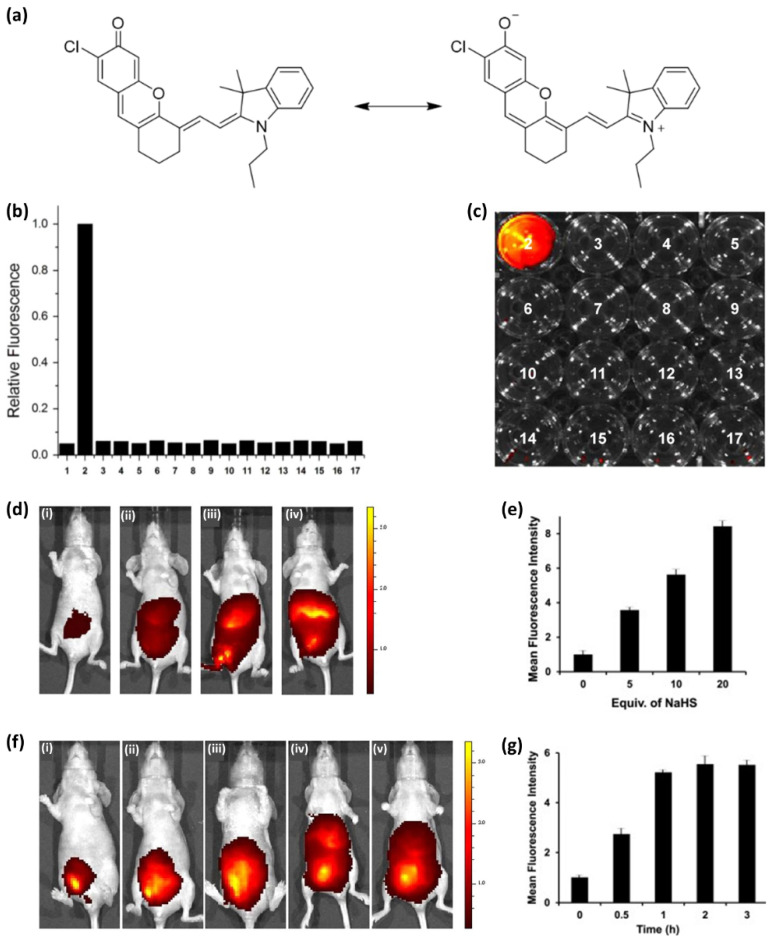
(**a**) Chemical structure of a synthetic H_2_S-activated compound. (**b**) Fluorescence enhancement of NIR-Az in the presence of thiol; reactive sulfur, nitrogen, and oxygen species; and ascorbic acid. Reactions of (1) 10 μM NIR-Az with (2) 200 μM NaHS, (3) 1 mM l-cysteine, (4) 1 mM dl-homocysteine, (5) 10 mM glutathione, (6) 200 μM Na_2_S_2_O_3_, (7) 200 μM Na_2_S_2_O_4_, (8) 200 μM Na_2_SO_3_, (9) 200 μM Na_2_SO_4_, (10) 200 μM NaHSO_3_, (11) 200 μM KSCN, (12) 200 μM H_2_O_2_, (13) 200 μM Angeli’s salt (NO^−^), (14) 200 μM NaNO_3_, (15) 200 μM NaNO_2_, (16) 200 μM ascorbic acid, and (17) 200 μM α-lipoic acid in PBS buffer (10 mM, pH 7.4, 30% acetonitrile, *v/v*) at 37 °C for 30 min (λex = 680 nm, λem = 720 nm). (**c**) Fluorescence images of 10 μM NIR-Az in the presence of thiol; reactive sulfur, nitrogen, and oxygen species; and ascorbic acid (λex = 675 nm, λem = 700 nm). (**d**) Representative fluorescence images of mice given i.p. injections of the NIR-Az probe (50 μM, in 20 μL of DMSO) and then injected with different amounts of NaHS: (i) 0, (ii) 5, (iii) 10, and (iv) 20 equivalents of NaHS (in 100 μL of PBS). (**e**) Quantification of the fluorescence emission intensity from the abdominal area of the mice of groups i–iv in (**d**). (**f**) Representative fluorescence images of mice administered an i.p. injection of the probe NIR-Az (50 μM, in 20 μL of DMSO) and then injected with 10 equivalents of NaHS (100 μM, in 100 μL of PBS). Images were taken after incubation of NaHS for (i) 0, (ii) 0.5, (iii) 1, (iv) 2, and (v) 3 h. (**g**) Quantification of the fluorescence emission intensity from the abdominal area of the mice of groups i–v in (**f**). Reprinted with permission from Ref. [232]. Copyright 2017, Elsevier.

These core structures have rich π electrons in common. In one remarkable study performed in 2017, Park et al. [232] utilized the π-abundant structure cyanine to detect hydrogen sulfide (Figure 4). Although hydrogen sulfide is a critical biological marker, few studies have focused on its detection in vivo. Park et al. [232] investigated the difference between absorption and emission peaks when their probe detected hydrogen sulfide. The probe displayed three absorption peaks at 550–680 nm; however, when the probe detected hydrogen sulfide, a new peak appeared at 700 nm. In the same vein, when the probe was exposed to hydrogen sulfide, the fluorescence intensity increased sharply at 720 nm. They further showed the highest occupied molecular orbital (HOMO), lowest unoccupied molecular orbital (LUMO) energy level, and bandgap; performed a selectivity test (Figure 4b); and obtained real-time fluorescence images in vivo using mice (Figure 4c).

### 3.1. UV Small-Molecule Probes

As an excitation energy, short-wavelength UV photons may induce bond cleavage, isomerization, or rearrangement reactions in organic and even inorganic molecules. Therefore, UV excitation presents several challenges, particularly in biological applications. For example, high-energy UV light has highly limited tissue penetration due to high optical scattering and absorption by endogenous chromophores, which can lead to sample overheating and in turn cause phototoxic or photoallergic reactions. 

**Figure 5:**
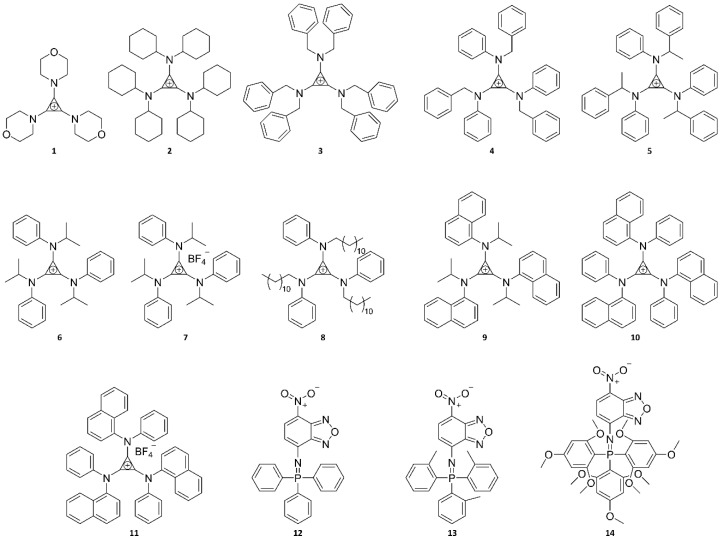
Chemical structure of organic probes (probes **1** to **14**) for bioimaging in the ultraviolet range.

**Figure 6:**
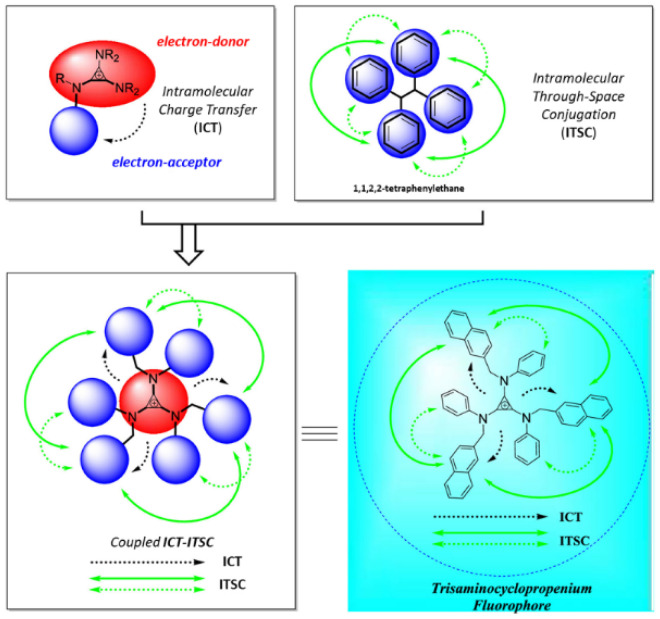
Schematic representation of intramolecular charge transfer (ICT) and intramolecular through-space conjugation (ITSC) and the coupling of these processes in trisaminocyclopropenium fluorophores. Reprinted with permission from Ref. [105]. Copyright 2020, American Chemical Society.

Examples of UV probes developed in recent studies are presented in Figure 5, which were mainly designed for the purposes of bioimaging DNA and HeLa cells in an in vitro context. This is considered to be due to the lower in vivo permeability of UV probes than NIR probes. Probes **1** to **11** consist of a cyclopropenium core and three primary amine arms, varying in size, conjugation, and structural flexibility. Remarkably, probes **10** and **11** have large Stokes shifts of ~180 nm, whereas most UV probes have small Stokes shifts (probes **1** to **9**: <l00 nm). Therefore, the use of probes **10** and **11** can achieve enhanced sensitivity by avoiding self-reabsorption effects. As illustrated in Figure 6, probes **10** and **11** have larger Stokes shifts due to the combination of two excitation processes: (i) ICT from the cyclopropenium core (electron donor) to the appended arms (electron acceptor) and (ii) intramolecular through-space conjugation among the appended arms. Nevertheless, it is important to note that molecular fluorophores suffer from ACQ when they are aggregated. To circumvent this effect, Montalti et al. [103] modified NBD (see Table 2) with triphenyphosphazene groups (probes **12**, **13**, and **14)**. Probes **13** and **14** successfully self-organized as highly bright and fluorescent nanoparticles with high colloidal stability in phosphate-buffered saline solution. Notably, in the aqueous phase, probe **13** nanoparticles displayed a brightness that was more than six orders of magnitude higher than that of the molecular counterpart in the organic solvent.

**Figure 7:**
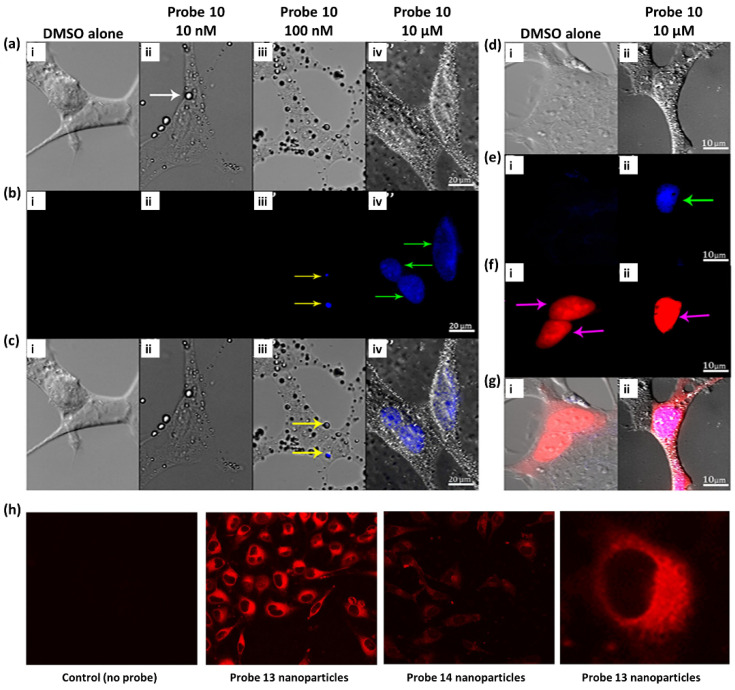
(**a**–**c**) Probe **10** fluorescently labels the nuclei of cells. HEK293 cells were imaged 5 min post-treatment in (**a**-i, **b**-i, **c**-i) 1% DMSO cell media, (**a**-ii, **b**-ii, **c**-ii) 1% DMSO and 10 nM probe **10** cell media, (**a**-iii, **b**-iii, **c**-iii) 1% DMSO and 100 nM probe **10** cell media, or (**a**-iv, **b**-iv, **c**-iv) 1% DMSO and 10 μM probe **10** cell media. (**a**-i, ii, iii, iv) Bright-field images. (**b**-i, ii, iii, iv) A 380 nm excitation channel and 450 nm emission fluorescence channel to image probe **10**. (**c**-i, ii, iii, iv) Merged images of bright-field and fluorescent images. Spherules (white arrows) are seen to form on the cell membranes at all concentrations tested of probe **10** (10 nM, 100 nM, and 10 μM) but fail to be observed when only DMSO was present. Several of these spherules produce significant amounts of blue fluorescence (yellow arrows), indicating the presence of probe **10**. The nuclei (green arrows) can be visualized following 10 μM treatment with probe **10**. (**d**–**g**) Validation of probe **10** for nuclear staining. HeLA cells were transfected with an mScarlet-NLS expression construct and imaged 5 min post-treatment in (**d**-i, **e**-i, **f**-i, **g**-i) 1% DMSO cell media or (**d**-ii, **e**-ii, **f**-ii, **g**-ii) 1% DMSO and 10 μM probe **10** media. (**d**-i, **d**-ii) Bright-field image. (**e**-i, **e**-ii) A 380 nm excitation channel and 450 nm emission fluorescence channel to image probe **10**. (**f**-i, **f**-ii) A 570 nm excitation channel and 600 nm emission fluorescence channel to image mScarlet-NLS. (**g**-i, **g**-ii) Merged images of all channels in the treatment condition. The nuclei (green arrows) can be visualized following 10 μM treatment, which overlaps with fluorescence being emitted from nuclei indicated by mScarlet-NLS (magenta arrows). Reprinted with permission from Ref. [105]. Copyright 2020, American Chemical Society. (**h**) Fluorescence signal of probe **13** and **14** nanoparticles in cells. HeLa cells grown on glass coverslips were incubated for 20 h with no nanoparticles (medium alone), probe **13** nanoparticles, or probe **14** nanoparticles and analyzed by fluorescence confocal microscopy with the same instrumental setting for comparison. Representative images are shown. In the case of probe **13** nanoparticles, cell signal distribution details are shown at higher magnification. Reprinted with permission from Ref. [103]. Copyright 2019, Frontiers Media SA.

These fluorescent probes belonging to the UV range have mainly been studied in the context of in vitro research and to determine their photophysical characteristics. First, Figure 7a–g shows a recent, notable example of the application of probe **10** for biolabeling [105]. The researchers hypothesized that this hydrophobic tris-amino cyclopropenium small-molecule phosphor could function as a membrane counterstain in mammalian cells. To test this hypothesis, probe **10**, which was dissolved in dimethyl sulfate oxide (DMSO), was added to HEK293 (human embryonic kidney) cells at concentrations of 10 nM, 100 nM, and 10 μM. Surprisingly, probe **10** formed membrane-related spheres outside the cell under all concentrations tested (Figure 7a(i)). Although the properties of these spheres were not examined in detail, it was assumed that the solution contains probe **10** based on fluorescence emission profiles (Figure 7a(ii),b(ii),c(ii)), consistent with the effects of probe **10**. Spheres containing probe **10** formed as a result of phase separation between the hydrophobic probe **10** and water-soluble culture medium, and they were associated with hydrophobic cell membrane lipid bilayers through hydrophobic interactions. In addition to the fluorescence emitted from some membrane-related spheroid cells, minimal fluorescence release was observed in cell membranes treated with 10 or 100 nM probe **10** solutions after excitation at 380 nm (Figure 7b(i),c(i)). However, cells treated with 10 μM of probe **10** showed bright fluorescence emissions at 450 nm in what appeared to be nuclei (Figure 7b(i),c(i)) and showed no morphological differences from those of controls before cell medium removal (Figure 7a–g). To verify this finding, HeLa cells (Henrietta Lacks, cervical cancer cell line) were implanted with DNA plasmids containing genetically encoded expression structures capable of inducing the cellular production of the red fluorescent protein mScarlet carrying orthogonal fluorescence sequences (mScarlet-NLS). Conversely, upon expression, mScarlet-NLS showed a distinct fluorescence excitation/emission profile compared with that of probe **10**, which can be used to visually decompose the two. After treating the HeLa cells expressing mScarlet-NLS with probe **10**, 450 nm fluorescence was clearly visualized as mScarlet fluorescence and co-localized in the cell nucleus (Figure 7d–g). In contrast, cells treated only with DMSO exhibited mScarlet fluorescence but did not exhibit fluorescence at 450 nm. Interestingly, in contrast to the hypothesis that probe **10** would function as a fluorescent membrane counterstain, specific labeling and localization of probe **10** to the cell nucleus were observed. This study adds to the burgeoning interest in cyclopropenium compounds and their use as fluorophores in bioimaging applications. 

Figure 7h exhibits a representative application example of probe **13** and **14** nanoparticles. Probe **13** and **14** nanoparticles were cultured with HeLa cells at a relatively low dose of 80 ng mL^−1^ at 37 °C to prove their effectiveness as fluorescent probes. After 20 h, confocal microscopy analysis showed that probe **13** nanoparticles provided an intense, structured signal within the cell cytoplasm, indicating endosomal internalization of the nanoparticles. Compared with those of probe **13** nanoparticles, probe **14** nanoparticles showed much weaker signals due to their intrinsically weak fluorescence characteristics. In addition, these probes showed that both probe **13** and **14** nanoparticles had no toxic effects at up to 1 g mL^−1^ in HeLa cells through separate viability assays, involving use of a colorimetric reagent (MTS) for the quantification of viable cells. In other words, the cellular experiments proved that probe **13** and **14** nanoparticles are suitable as fluorescent contrast agents for bioimaging owing to their good biocompatibility. These results further demonstrated that the rational design of a molecular precursor is fundamental for producing stable and intensely bright aggregate nanoparticles. 

### 3.2. NIR Small-Molecule Probes

**Figure 8:**
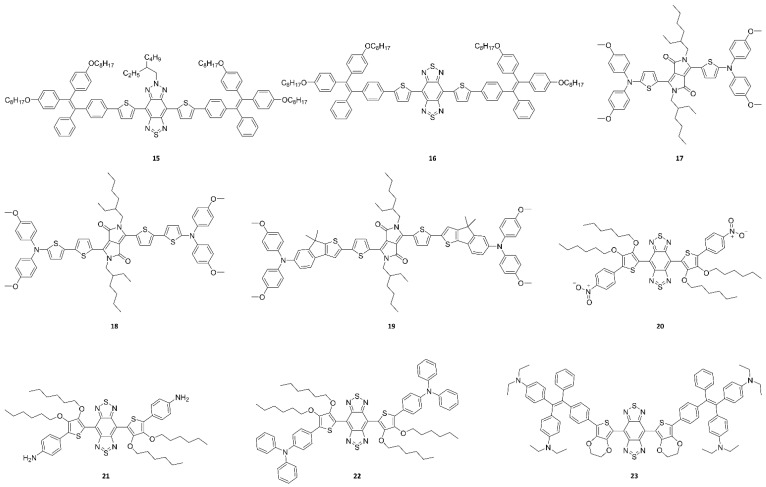
Scheme of chemical structures (probes **15**–**23**) for bioimaging in the near-infrared range.

Compared to UV light, NIR light can penetrate deeper into tissues without inducing any photochemical damage. In addition, NIR light sources are usually cheaper, more common, and more accessible to non-specialist end users. Figure 8 exhibits molecular probes for which their fluorescence emissions belong to the NIR region, which has been reported in recent studies [47,111,112,116]. As described above, probes belonging to the NIR region are more suitable for in vivo applications because they more easily penetrate cells than probes belonging to the UV region. Although the concept of small molecule-based NIR fluorophores is simple, their synthesis and purification require multiple steps and mostly result in low yields. Therefore, optimizing the procedures for the design and synthesis of the molecular probes remains a challenge for organic chemists.

The major strategies for designing NIR molecular probes can be summarized as follows. NIR-I probes have been mostly obtained with a donor–acceptor (D–A) system. The combination of strong electron donors and acceptors in a molecule may lower the bandgap energy, resulting in NIR-I probes with emission wavelengths of ~900 nm. Moreover, further introducing a second strong donor in the D–A architecture allows yielding a symmetrical donor–acceptor–donor (D–A–D) architecture. The strong electron-donating groups adjacent to the central electron acceptor reduce the energy gap between hybridized HOMO/LUMO levels, which drives the fluorescence emission into the NIR-II region (emission wavelengths longer than 1000 nm). Representatively, BBTD, which is a strong electron-deficient unit, has been widely used as an acceptor. However, owing to the strong ICT effect, BBTD-based fluorophores suffer from low quantum yields in aqueous solution. Therefore, there is a demand for the development of new acceptor units to afford more alternative design strategies of NIR-II molecular probes. The combination of various spacers and electron donors/acceptors can provide unique opportunities to expand the library of small molecule-based NIR-II fluorophores. Table 3 summarizes the absorption peak, fluorescence emission peak, and Stokes shifts of the small-molecule probes that have been recently reported in bioimaging studies. Most of these studies have calculated the HOMO/LUMO levels and the resulting bandgap energies through density functional theory, which are also given in Table 3.

### 3.3. Nanoparticulate Small-Molecule Probes

**Figure 9:**
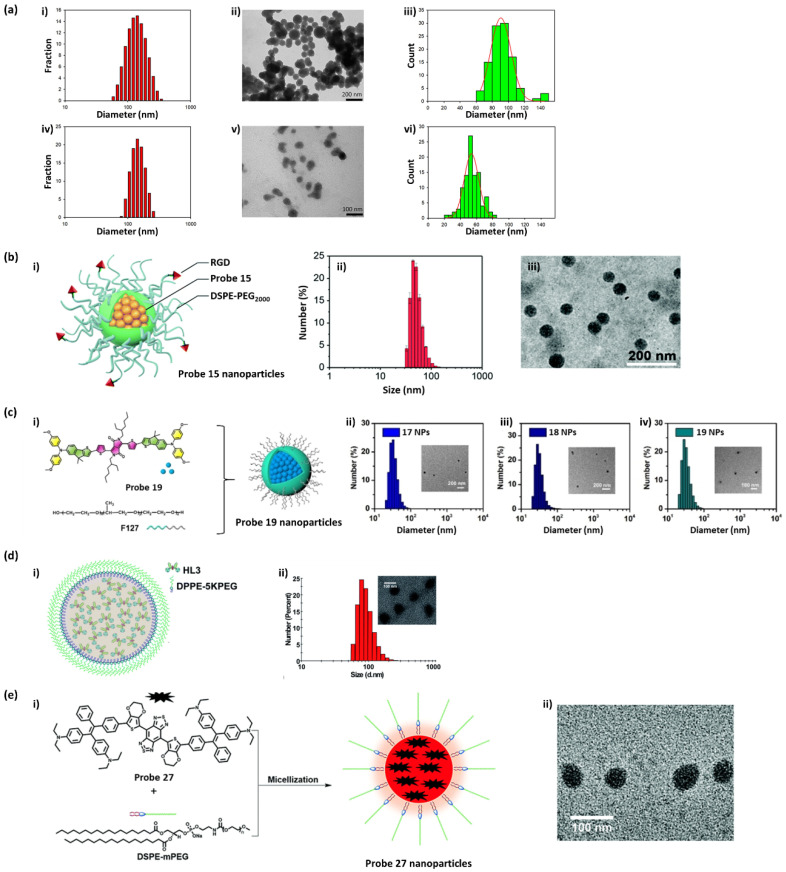
(**a**-i) Size distribution obtained by dynamic light scattering (DLS) analysis of nanoparticles from probe **13**. (**a**-ii) Representative transmission electron microscopy (TEM) image of probe **13** nanoparticles. (**a**-iii) Size distribution resulting from the analysis of the TEM images of probe **13** nanoparticles. (**a**-iv) Size distribution obtained by DLS analysis of probe **14** nanoparticles. (**a**-v) Representative TEM image of probe **14** nanoparticles. (**a**-vi) Size distribution resulting from the analysis of TEM images of probe **14** nanoparticles. Reprinted with permission from Ref. [103]. Copyright 2019, Frontiers Media SA. (**b**–i) Schematic illustration of probe **15** nanoparticles. (**b**-ii) TEM images of probe **15** nanoparticles. (**b**-iii) Size measurement of probe **15** nanoparticles in water using DLS. Reprinted with permission from Ref. [111]. Copyright 2021, Royal Society of Chemistry. (**c**-i) Schematic illustration of probe **19** encapsulating nanoparticles by Pluronic F127. The DLS spectrum of (**c**-ii) probe **17** nanoparticles with F127, (**c**-iii) probe **18** nanoparticles with F127, and (**c**-iv) probe **19** nanoparticles with F127 (inset: TEM image of nanoparticles). Reprinted with permission from Ref. [116]. Copyright 2021, John Wiley and Sons. (**d**-i) Formation of probe **22** nanoparticles via nanoprecipitation. (**d**-ii) DLS and TEM images of probe **22** nanoparticles; scale bar: 100 nm. Reprinted with permission from Ref. [47]. Copyright 2021, Royal Society of Chemistry. (**e**-i) Schematic illustration of the preparation method of NIR-II AIE nanoparticles (probe **23** nanoparticles) via micellization. (**e**-ii) Representative TEM image of probe **23** nanoparticles. Scale bar: 100 nm. Reprinted with permission from Ref. [112]. Copyright 2021, Royal Society of Chemistry.

To improve in vivo bioavailability, nanoparticulate molecular fluorophores can be prepared by physically encapsulating or chemically conjugating/modifying them with biocompatible polymers and peptides. Figure 9 provides examples of preparing nanoparticulate small molecule-based probes from several studies. 

First, Figure 9a exhibits the morphology of nanoparticles prepared from probes **13** and **14** using a nanoprecipitation method [103]. Transmission electron microscopy (TEM) images showed that the probe **13** and **14** nanoparticles have diameters of 91 ± 13 and 54 ± 9 nm, respectively. The diameter measured for probe **13** nanoparticles by TEM was consistent with the hydrodynamic diameter measured by dynamic light scattering (DLS). However, a large difference between the TEM and DLS diameters was observed in the case of probe **14** nanoparticles, indicating partial inter-nanoparticle aggregation in aqueous solution. The hydrodynamic diameter is much more important in practical applications because it more precisely reflects how the nanoparticles move in a fluid. Next, using probes **15** and **16**, water-dispersible nanoparticles were also fabricated using a modified nanoprecipitation method (Figure 9b) [111]. TEM images indicated that these nanoparticles are spherical in shape with a narrow particle size distribution. DLS analysis revealed that the nanoparticles with hydrodynamic diameters of 52.5 ± 3.4 and 55.7 ± 4.2 nm were prepared from probes **15** and **16**, respectively. To improve biocompatibility, probes **17**, **18,** and **19** were encapsulated into nanoparticles by a pluronic surfactant (F127) through nanoprecipitation (Figure 9c) [116]. The pluronics correspond to polyethylene oxide-polypropylene oxide-polyethylene oxide-based triblock copolymers, which are available to serve as non-ionic surfactants with varying compositions and hydrophilic–lipophilic balance. In particular, Pluronic F127 is the most popular pluronic for various biological applications because it is approved by the FDA as an excipient in oral, ophthalmic, and topical medicinal formulations. TEM revealed that the morphologies of the nanoparticles fabricated from probes **17**, **18**, and **19** were spherical. The DLS diameters of the nanoparticles were found to be approximately 30 nm. During storage at 4 °C for 20 days, no significant variations of the zeta potential and polymer dispersity index were observed, indicating the excellent stability of these nanoparticles. 

In probes **20**−**22**, the substituted hexyloxy chains serve as a strong donor, and other electron-withdrawing or electron-donating groups such as nitrobenzene (probe **20**), aminobenzene (probe **21**), and triphenylamine (probe **22**) were also introduced as substituted groups into the probes [47]. Importantly, in probe **22**, 3,4-bis(hexyloxy)thiophene and triphenylamine serve as the first donor and second donor, respectively, for AIE. Probe **21** and **22** nanoparticles with high monodispersity and homogeneity were also prepared using a nanoprecipitation method with the aid of a DPPE-5KPEG amphiphile (Figure 9d). Representatively, probe **22** nanoparticles were characterized for bioimaging applications using TEM and DLS, showing an average size of ~90 nm and a dynamic size of ~120 nm. Probe **23**, based on BBTD and TPE, shows NIR-II fluorescence with AIE characteristics [112]. To illustrate the feasibility of probe **23** for bioimaging application, similarly to probes **21** and **22**, water-soluble and biocompatible nanoparticles were fabricated with a nanoprecipitation method using an amphiphile (DSPE-PEG5000) (Figure 9e). Briefly, a mixture of probe **23** and THF was rapidly added into an aqueous DSPE-PEG5000 solution in an ice bath under continuous sonication; then, the remaining THF and DSPE-PEG5000 were carefully removed. The resulting nanoparticles exhibited high monodispersity and homogeneity with an average particle size of ~50 nm, as measured by TEM, and a hydrodynamic diameter of ~60 nm, as measured by DLS.

### 3.4. In Vivo Bioimaging

**Figure 10:**
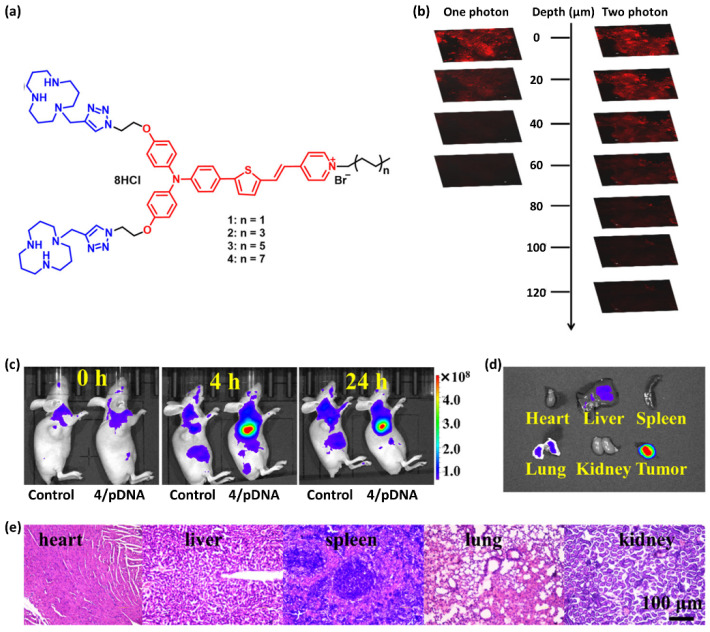
(**a**) Structures of probe molecules consisting of three parts: two polar [12]aneN_3_-triazole heads (blue), a luminogen core (red), and a long hydrophobic tail (black). (**b**) Ex vivo one-photon or two-photon images of the tumor tissue injected with [12]aneN_3_-modified TPA derivatives/pUC18 DNA. (**c**) In vivo imaging at different time points after intratumor injection with [12]aneN_3_-modified TPA derivatives/pUC18 DNA. (**d**) Ex vivo biodistribution of various organs and tumor tissue from mice 24 h post-injection with [12] aneN_3_-modified TPA derivatives/pUC18 DNA. (**e**) Major organs harvested from the mice after being intratumorally injected with [12]aneN_3_-modified TPA derivatives/pUC18 DNA for 21 days. Reprinted with permission from Ref. [234]. Copyright 2021, American Chemical Society.

To date, many strategies for in vivo bioimaging using small molecule-based probes have been developed to obtain information about biological functions and diseases [233]. As an example, Xu and co-workers [234] reported a two-photon-triggered NIR fluorescence technique for cancer theranostics (imaging and photodynamic/gene therapy) using small-molecule probes with large π-conjugated TPA derivatives and a lipophilic tail (Figure 10). Notably, the unique molecular architecture allowed TPA-based probes to acquire NIR AIE characteristics, large Strokes shifts, and intense two-photon excitation fluorescence. In addition, the TPA-based probe molecules assembled together with DNA to form nanoparticles, triggering the AIE property. Consequently, TPA-based probes showed high two-photon excitation on four different types of cell lines in the absence/presence of serum and were capable of tracking the transfection process and monitoring the real-time in vivo biodistribution with high resolution and deep penetration. In vivo bioimaging using the TPA-based probe with DNA to tumor-bearing nude mice displayed bright fluorescence over time, indicating that the probe has excellent tumor retention properties as well as long-term biocompatibility, demonstrating the promise of two-proton fluorescence using the TPA-based probe for bioimaging and theranostics. 

**Figure 11:**
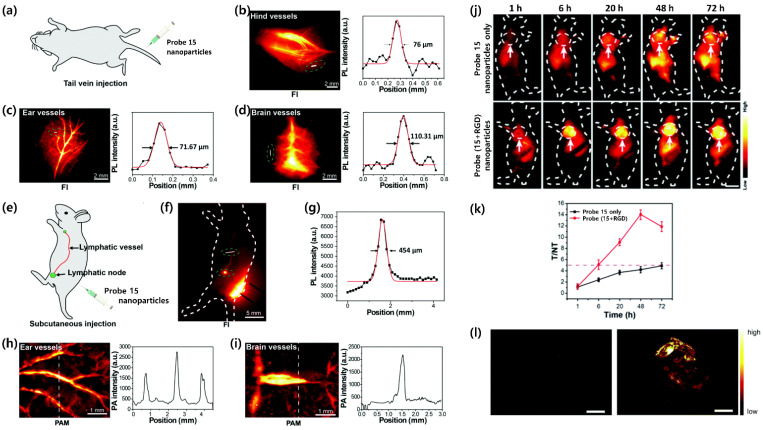
Probe **15** nanoparticle-based imaging in vivo. (**a**) Schematic illustration of intravenous injection of the probe nanoparticles through the tail vein of a mouse. (**b**–**d**) Representative NIR-II fluorescence images of the hindlimb vasculature (**b**), ear vessels (**c**), and brain vessels (**d**) in the mouse upon intravenous injection of 100 mg of the probe nanoparticles. (**e**) Schematic illustration of subcutaneous injection of the probe nanoparticles for lymphatic imaging. (**f**) Representative NIR-II fluorescence image of the lymphatic vessels and lymph nodes in a mouse injected with the nanoparticles. (**g**) Cross-sectional fluorescence intensity profile (and Gaussian fit) along the red line, circled in green, in panel (**f**). PAM imaging of the ear vessels (**h**) and brain vessels (**i**) from a mouse injected with the probe nanoparticles through the tail vein. (**j**) Representative NIR-II FLI in 143B tumor-bearing mice using the probe-based nanoparticles over 72 h under excitation by an 808 nm diode laser (140 mW cm^−2^); filter: 1000 nm LP. Arrows indicate the subcutaneous tumor (*n* = 4 per group). Scale bar: 5 mm. (**k**) T/NT ratios in probe nanoparticle-based 143B tumor imaging over 72 h. Data are plotted as mean and standard deviation (*n* = 4). (**l**) Three-dimensional volume rendering of photoacoustic images (excitation at 730 nm) of the 143B tumor in a mouse 48 h post-injection of the probe nanoparticles without (left) or with (right) RGD peptide decorated on the surface. Scale bar = 2 mm. Reprinted with permission from Ref. [111]. Copyright 2021, Royal Society of Chemistry.

There are also several remarkable studies on in vivo bioimaging using small molecules, as described above. Figure 11 summarizes the results of a recent study that investigated NIR-II fluorescence imaging using small-molecule probe **15**. To evaluate the potential of probe **15** nanoparticles for imaging of the animal circulatory system, Li and colleagues [111] performed vascular imaging in mice through both NIR-II fluorescence and NIR-I photoacoustics using an animal fluorescence imaging system and photoacoustic microscopy (PAM), respectively. Real-time video-rate NIR-II fluorescence imaging of the vascular networks in the ears, hindlimbs, and brain was performed on C57BL/6J mice following intravenous injection of probe **15** nanoparticles (1000 LP). At 10 min after administration of the nanoparticles (50 mg per mouse), the vasculature from the hindlimbs, ears, and brain could be clearly visualized with high resolution under 785 nm excitation at a power density of 100 mW cm^−2^. The cross-sectional fluorescence profiles of the representative vessels were plotted with the physical location along the white lines. The results suggested that the fluorescence profiles from the blood vessels of the hindlimbs, ears, and brain have a full width at half maximum (FWHM) of 76.0, 71.6, and 110.3 μm, respectively, indicating the high sensitivity and spatial resolution. Cerebral vessel imaging was also performed under an intact skull. Upon subcutaneous injection of probe **15** nanoparticles through the flank of a mouse, the adjacent lymph node to the injection site was lit up, providing a high-resolution image of the lymphatic vessel with an FWHM of 454.0 μm (Figure 11e–g). PAM was employed to further demonstrate the high spatial and temporal resolution for in vivo vasculature imaging provided by probe **15** nanoparticles. Nude mice were anesthetized and placed on the imaging stage before imaging blood vessels in the ear and brain. Upon intravenous administration, the blood vessels were lit up and clearly observed upon 730 nm pulse laser excitation (Figure 11h–i). PAM indicated that the FWHM of the ear blood vessels was 223.0 μm. In brain vessel imaging upon scalp removal, both the transverse sinus and superior sagittal sinus were clearly visualized (Figure 11i). In addition, the inferior cerebral veins also emitted photoacoustic signals upon probe **15** nanoparticle injection, whereas they were nearly undetectable before probe **15** nanoparticle injection. The tumor-targeting ability of the nanoparticles was then examined in nude mouse models subcutaneously inoculated with 143B osteosarcoma cells and PC3 prostate carcinoma cells (Figure 11j–l). For each type of tumor, two groups of tumor-bearing mice were used to confirm the targeting ability of probe **15** nanoparticles comprising Arg–Gly–Asp peptide (RGD) (*n* = 4 per group). The first group of mice was injected with probe **15**–RGD nanoparticles, while another control group of tumor-bearing mice was treated with probe **15** nanoparticles. The NIR-II fluorescence signals from the tumor sites gradually increased and reached a maximum at 48 h post injection of probe **15**–RGD nanoparticles in both tumor-bearing mouse groups (Figure 11j,k). However, the signals from tumor sites in mice injected with probe **15** nanoparticles revealed only limited enhancement within the test period. Additionally, the fluorescence intensity from the tumor sites of the mice injected with probe **15**–RGD nanoparticles was higher than that from the control group at all time points. Quantification suggested that the maximum average signal-to-background (T/NT) ratios of probe **15**–RGD nanoparticle-treated mice were ~14.2 and ~8.1 in the 143B and PC3 tumor models, respectively, while those of the corresponding probe **15** nanoparticle-treated mice were only 5.0 and 4.9 at 72 h post injection. The 143B tumor-bearing mice were sacrificed to collect the vital organs and tissues for imaging. The quantitative analysis of ex vivo fluorescence imaging results suggested that all these organs and tissues from mice injected with probe **15**–RGD nanoparticles emitted fluorescence with different signal intensities. In particular, the injected probe **15**–RGD nanoparticles were mainly distributed in the tumor, liver, spleen, and lymph node. The skin sample also showed fluorescence signals, indicating the retention of probe **15**–RGD nanoparticles due to high binding affinity with the integrin AVB3 expressed in the skin tissues. In addition to fluorescence imaging, photoacoustic tomography was also performed to further confirm the overall targeting effect of probe **15**–RGD nanoparticles at tumor sites (Figure 11l). The photoacoustic signal intensity at the tumor site from a probe **15**–RGD nanoparticle-treated mouse was also higher than that of a probe **15** nanoparticle-treated mouse, which was consistent with the results obtained from NIR-II fluorescence imaging. These results successfully demonstrated the high potential of probe **15** nanoparticles in dual-modality bioimaging applications. Thus, by taking advantage of the high sensitivity of NIR-II fluorescence imaging, a clear visualization of tumor margins in a two-dimensional view was successfully achieved.

**Figure 12:**
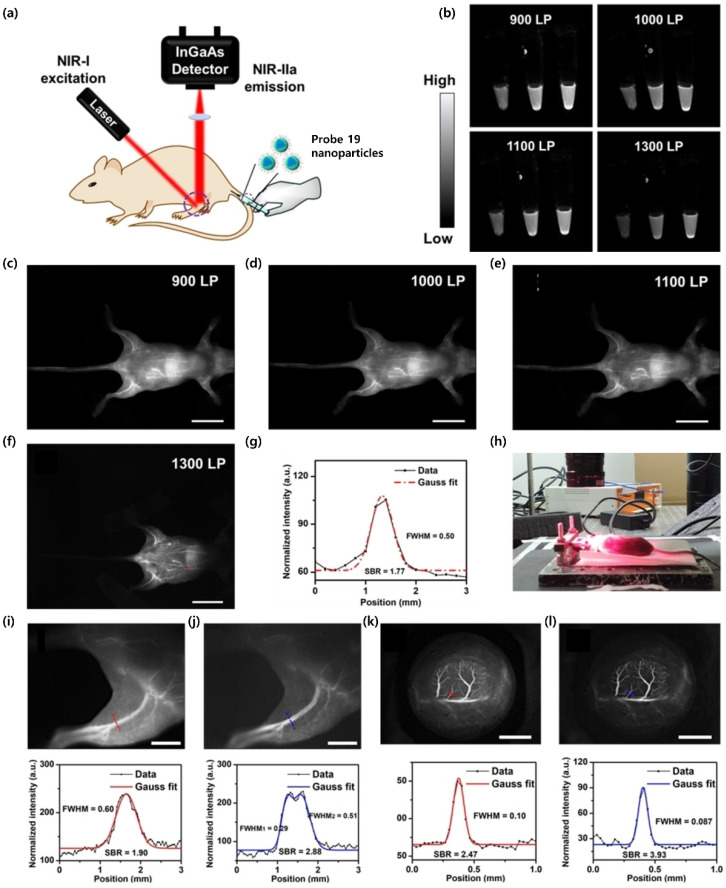
Probe **19** nanoparticle-based imaging in vivo. (**a**) Schematic illustration of NIR-II fluorescence imaging of the hindlimb vasculatures in BALB/c nude mice using a wide-field imaging system. (**b**) Comparison of NIR-II fluorescence signals at different concentrations with different LP filters (0.1, 0.25, and 0.5 mg mL^−1^). (**c**–**g**) NIR-II images of the whole-body vessels of mice after intravenous injection of the probe **19** nanoparticles with F127 under irradiation with a 690 nm laser (200 μL, 0.5 mg mL^−1^). Scale bar = 2 cm. (**h**) Photographs of the devices and instruments used in hindlimb and brain blood vessel imaging experiments. (**i**) NIR-II and (**j**) NIR-IIa fluorescence images of the hindlimb blood vessels of nude mice using 1100 nm and 1300 nm LP filters, respectively. Scale bar = 5 mm. (**k**) NIR-II and (**l**) NIR-IIa fluorescence images of the brain blood vessels during craniotomy in C57BL/6 mice using 1100 nm and 1300 nm LP filters, respectively. (Scale bar = 3 mm). Reprinted with permission from Ref. [116]. Copyright 2021, John Wiley and Sons.

Next, as shown in Figure 12a, photoluminescence angiography was successfully realized as a non-radioactive imaging strategy by intravenously injecting probe **19** nanoparticles with Pluronic F127 [116]. NIR-II (900–1700 nm) and NIR-IIa (1300–1400 nm) fluorescence images of whole-body blood vessels in mice were acquired with different filters (900–1300 nm) after intravenous injection of the probe nanoparticles (200 μL, 0.5 mg mL^−1^). With an increase in the wavelengths of the long-pass (LP) filter, the spatial resolution of images considerably improved. Higher-resolution angiography images of the mouse were obtained in the NIR-IIa region with a Gaussian-fitted FWHM of 0.50 mm and SBR of 1.77. The distance between the camera and camera lens was increased to acquire magnified images. NIR-IIa fluorescence imaging was then further applied to the hindlimb blood vessels. As displayed in Figure 12i, the NIR-II fluorescent image (1100 nm LP filter) displayed the clear morphology of blood vessels. To evaluate the quality of NIR-II fluorescence imaging, a line was drawn across the femoral vessels, and Gaussian-fitted analysis was performed. The FWHM of the femoral vessels was 0.60 mm, and SBR was 1.90. The femoral artery and femoral vein, which are very close and thus difficult to distinguish with the 1100 nm LP filter, were clearly observed in the NIR-IIa region (1300 nm LP filter) with higher resolution (femoral artery, FWHM1 = 0.29 mm; femoral vein, FWHM2 = 0.51 mm; SBR = 2.88). In addition, cerebral blood vessel imaging was further carried out in mice after performing a craniotomy. As presented in Figure 12k, due to the high SBR of bioimaging in the NIR-II region (1100 nm LP), the cerebral blood vessels were clearly imaged by an InGaAs detector (FWHM = 100 μm, SBR = 2.47) after the intravenous injection of the probe nanoparticles (200 μL, 500 μg mL^−1^). In NIR-IIa fluorescence images (1300 nm LP) of the cerebral blood vessels, the same cerebrovasculature was observed at higher spatial resolution (FWHM = 87 μm, SBR = 3.93), which further confirmed that fluorescence imaging in the NIR-IIa region can render better clarity and resolution compared with that in the NIR-II region.

**Figure 13:**
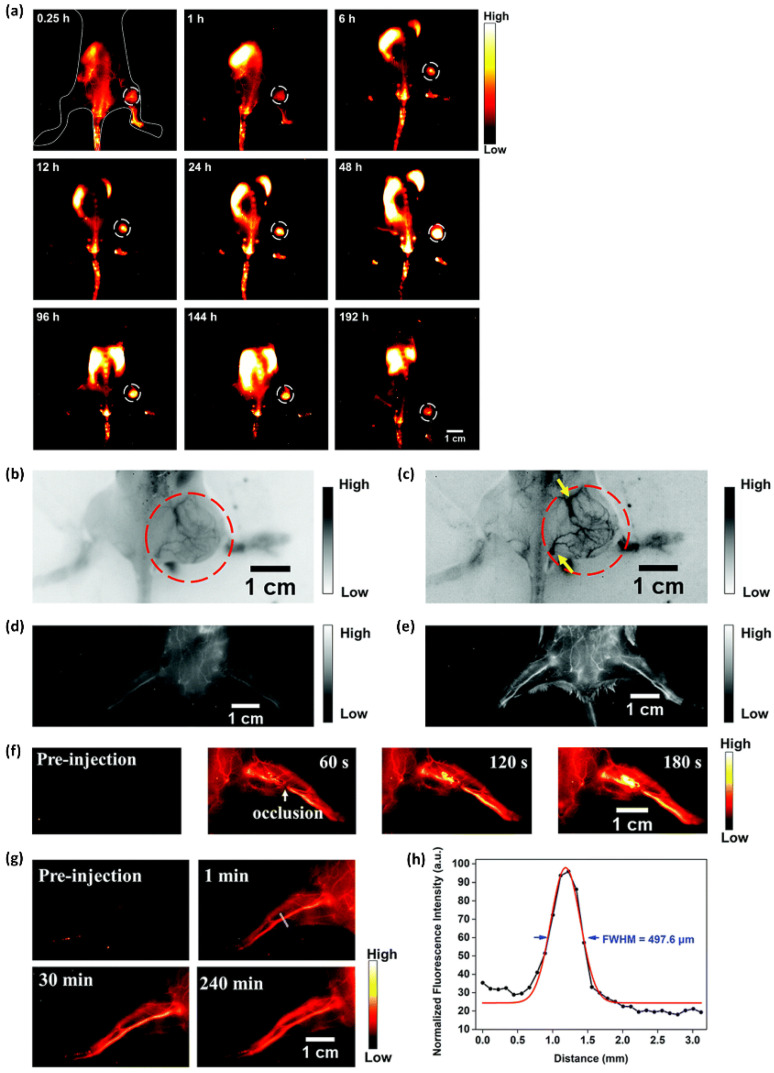
Probe **23** nanoparticle-based imaging in vivo. (**a**) Representative in vivo long-term NIR-II fluorescence images (808 nm excitation, 90 mW cm^−2^; 1000 nm LP, 100 ms; *n* = 3) of the 4T1 breast tumor model at different time points after tail vein injection of the probe nanoparticles (0.2 mL, 10 mg kg^−1^). The white circles indicate the location of the 4T1 tumor. Scale bar: 1 cm. (**b**,**c**) In vivo visualization of tumor-feeding vessels. NIR-II tumor blood vessel fluorescence images of the 4T1 breast tumor-bearing mice obtained at 2 min post tail vein injection of the probe nanoparticles (0.2 mL, 10 mg kg^−1^) with (**b**) 1000 nm (200 ms, 808 nm excitation, 90 mW cm^−2^) and (**c**) 1250 nm (800 ms, 808 nm excitation, 180 mW cm^−2^) LP filters, respectively. The red circles indicate the tumor location, and yellow arrows indicate the tumor-feeding arteries. (**d**,**e**) NIR-II fluorescence images of the hindlimb vessels in C57BL/6 mice at 2 min after tail vein injection of the probe nanoparticles (0.2 mL, 15 mg kg^−1^) with (**d**) 1000 nm (60 ms, 808 nm excitation, 90 mW cm^−2^) and (**e**) 1250 nm (500 ms, 808 nm excitation, 180 mW cm^−2^) LP filters, respectively. (**f**) In vivo NIR-II fluorescence images (1250 nm LP, 400 ms, 808 nm excitation, 180 mW cm^−2^) of incomplete left hindlimb ischemia pre-injection, and 60 s, 120 s, and 180 s after injection of the probe nanoparticles; the white arrow indicates the occlusion site in the femoral artery. (**g**) Intravital long-term hindlimb vasculature NIR-II imaging (1250 nm LP, 400 ms, 808 nm excitation, 180 mW cm^−2^) from 0 min to 240 min after tail vein injection of the probe nanoparticles. Scale bar (**b**–**g**): 1 cm. (**h**) The vessel FWHM width based on the cross-sectional intensity profile measured along the white line in (**g**) (1 min) with the peak fitted to Gaussian functions (the red curve is the Gaussian fit to the profile). Reprinted with permission from Ref. [112]. Copyright 2019, Royal Society of Chemistry.

NIR-II fluorescent probe **23** nanoparticles with the AIE property were also available for visualizing tumor-feeding blood vessels, long-term hindlimb vasculature, and incomplete hindlimb ischemia, as well as long-term breast tumor imaging. Figure 13 summarizes the data of in vivo NIR-II fluorescence imaging for breast tumors and tumor-feeding blood vessels using probe **23** nanoparticles [112]. The in vitro cellular endocytosis of probe **23** nanoparticles was examined using 4T1 breast cancer cells. The NIR-II fluorescence intensity of the 4T1 cells incubated with probe **23** nanoparticles revealed explicit time dependence, indicating that probe nanoparticles can permeate cells via the cellular membrane. To examine the ability of the probe nanoparticles to diagnose breast cancer early, a mouse model of breast cancer was established in which 4T1 breast cancer cells (approximately 2 × 10^6^) were subcutaneously injected into the right leg of female BALB/c mice. NIR-II fluorescence imaging of 4T1 breast tumors was conducted by intravenously injecting the probe nanoparticles (0.2 mL, 10 mg kg^−1^) into 4T1 tumor-bearing mice (*n* = 3) through the tail vein when the tumors reached ~60 mm^3^ in 1 week. The fluorescence intensity (1000 nm LP, 100 ms) in tumors reached the highest level at 48 h post-injection under 808 nm excitation with a power density of 90 mW cm^−2^ (Figure 13d). For up to 8 days post-injection, the NIR-II fluorescence signal of tumors was very strong and clearly delineated the normal tissues, demonstrating the suitability of the probe nanoparticles for long-term tumor imaging and real-time image-based monitoring in solid tumor therapy. An ex vivo biodistribution study was also carried out to evaluate the distribution of the probe nanoparticles in the major organs. High accumulation was observed in the liver and spleen, indicating that the probe nanoparticles would be cleared through the hepatobiliary system. 

The probe **23** nanoparticles were then intravenously injected into 4T1 tumor-bearing BALB/c mice (*n* = 3) to evaluate their feasibility as an NIR-II contrast agent for the in vivo imaging of the tumor-feeding blood vessels. At 2 min post-injection, the tumor vasculature was successfully visualized using an InGaAs camera with 1000 nm (Figure 13b, 200 ms exposure time) and 1250 nm (Figure 13c, 800 ms exposure time) LP filters under 808 nm laser excitation. NIR-II fluorescence images of the 4T1 tumor blood vessels showed a more distinct vascular network with the 1250 nm LP filter than with the 1000 nm filter, due to both reduced biological auto-fluorescence and photon scattering in the longer-wavelength NIR-II region. Moreover, a similar image quality via different LP filters was demonstrated in the hindlimb vasculature of C57BL/6 mice (Figure 13d,e). Surgery was conducted to induce incomplete hindlimb ischemia to mimic the NIR-II fluorescence imaging-guided vascular embolization procedure, and the occlusion site of blood supply vessels was precisely monitored by NIR-II imaging using the probe nanoparticles (Figure 13f). The capability of long-term vasculature imaging using the probe nanoparticles was then examined. As exhibited in Figure 13g, the vasculature was still clearly visualized through NIR-II imaging at 4 h post-injection. Furthermore, the resolution (497.6 mm) of the hindlimb vasculature calculated through the Gaussian-fitted FWHM confirmed the capability of the probe nanoparticles to precisely map vascular vessels with NIR-II imaging (Figure 13h).

## 4. Polymer Probes for Bioimaging

Table 4 introduces key chemical units that have been frequently used in the design of polymeric luminescent probes. Benzothiadiazole (BTD) is a heteroaromatic compound containing S and N atoms. BTD derivatives, including BBTD, with photophysical and electron-withdrawing properties have potential applications in organic light-emitting diodes, photovoltaic cells, and organic field-effect transistors [198,202,235,236]. Pentiptycene is a member of the iptycene family characterized by five benzene rings fused to a rigid H-shaped scaffold with more than three times the internal free volume of the triptycene unit [237]. The structure of pentiphene has been widely studied in microporous organic polymers, MOFs, and COFs due its great potential to increase microporosity and molecular adsorption capacity. Triazoles with a 5-membered ring of two carbon atoms and three nitrogen atoms can be divided into 1,2,3-triazole and 1,2,4-triazole and are easily obtained [238]. Triazole derivatives can act as bioisosteres of amides, esters, and carboxylic acids. Therefore, triazole derivatives have various pharmacological properties such as antibacterial, anticancer, and anti-tuberculosis activities. Moreover, their solubility and binding strength can be improved through various non-covalent interactions between the derivative and the biomolecule target. Fluorene, an ortho-fused tricyclic hydrocarbon, has a violet fluorescence [239]. Fluorene and its derivatives have been widely used as key materials in various biomedical and optoelectronic applications. The fluorene unit is frequently used to enhance molecular rigidity and reduce the intermolecular interaction in molecular probes. Spirobifluorene comprises two fluorene units connected through the spiro-carbon atom [240,241]. This unique spatial configuration induces strong rigidity with enhanced stability, preventing the oxidation process occurring at position 9 of the fluorene ring. Similarly to fluorene, spirobifluorene can also be chemically modified with donor and/or acceptor groups to tune the emission of the system. 

Quinoxaline, also called benzopyrazine, is a well-known nitrogen-containing heterocyclic compound composed of benzene and pyrazine rings [242,243]. The quinoxaline unit, which contains electron-withdrawing nitrogen atoms, is highly electron-deficient and, thus, may serve as an efficient electron acceptor. Boron-dipyrromethene (BODIPY) is an important class of versatile fluorophores with remarkable optical properties [244,245,246]. The use of the BODIPY unit may offer several advantages such as (i) strong absorption and photoluminescence throughout the UV–vis region that can be tuned toward the NIR region; (ii) a relatively high molar extinction coefficient and photoluminescence quantum yield; (iii) photochemical and thermal robustness, high electron affinity, and ease of synthetic functionalization; and (iv) tunable electronic and photonic properties. 

Porphyrins are a class of macrocycles consisting of four pyrrole rings conjugated through methine bridges [247,248]. Due to their large π-aromatic systems, porphyrins possess excellent chemical and thermal stability as well as distinct photophysical and electrochemical properties, which can be regulated by substituting the functional groups and the coordinated metal ions. In addition to the coordination of metal ions at the porphyrin center, the periphery of porphyrins can also be bound to metal ions if the peripheral rings are properly functionalized. Intricate binding modes enable porphyrins to form desirable molecular cages or a solid framework. The ability of porphyrins and their derivatives to strongly absorb light at wavelengths near 400 nm has been widely exploited for various light-harvesting applications. Lastly, methylene blue, a blue cationic thiazine dye, is widely used in various industrial areas such as in food, cosmetics, textiles, pharmaceuticals, and medicine [249,250]. Remarkably, methylene blue has received FDA approval for the treatment of methemoglobinemia. Methylene blue has an absorption peak at 665 nm with a shoulder at 610 nm and an emission at ~690 nm. 

### 4.1. UV/NIR Polymer Probes

**Figure 14:**
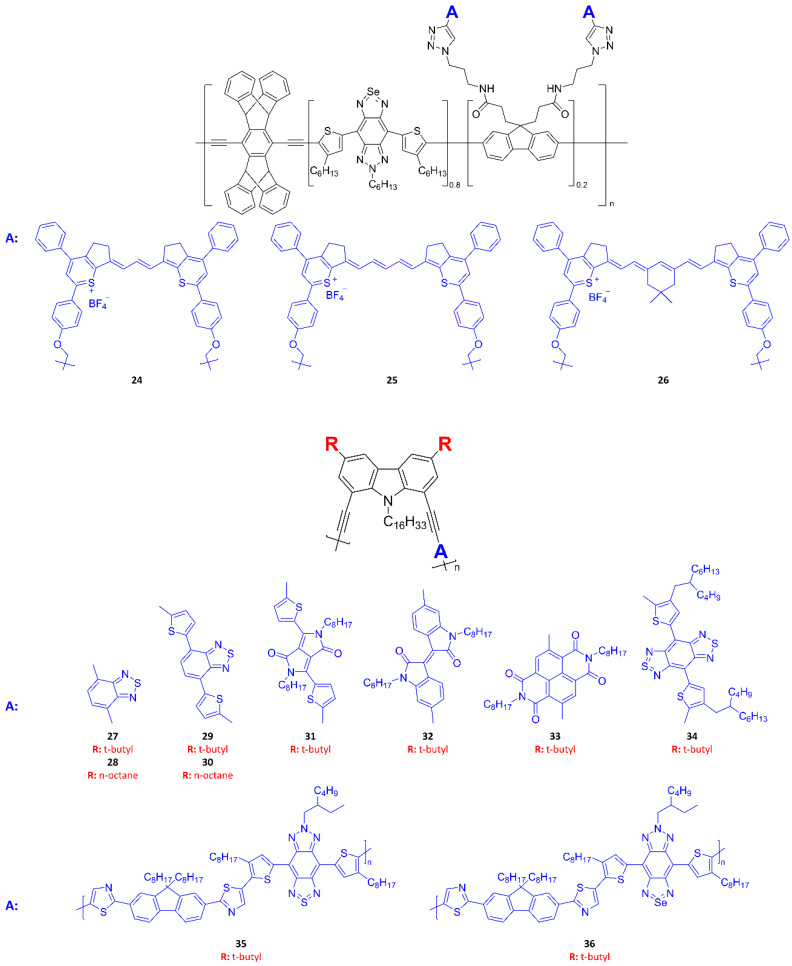
Chemical structures of the polymer probes constituting polymer dots, via the strategy of tuning the alkyl structure to change their optical properties.

**Figure 15:**
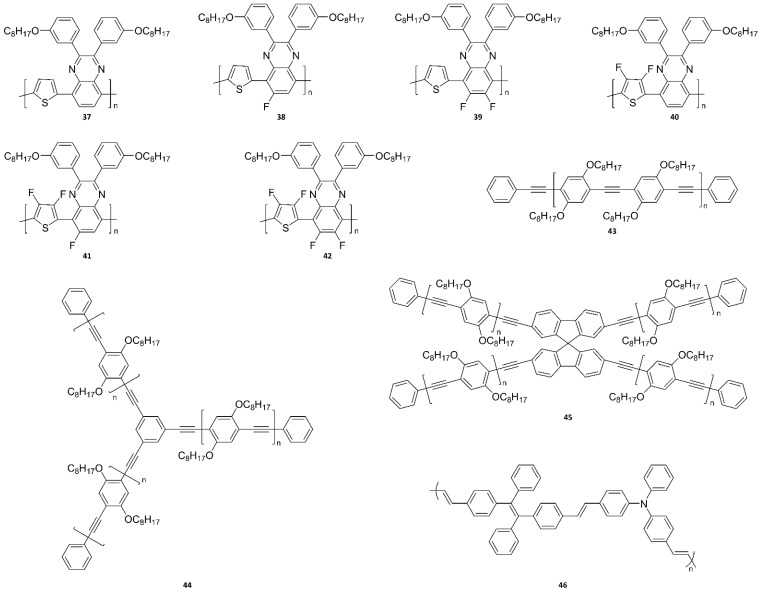
Chemical structures of the polymer probes constituting polymer dots.

**Figure 16:**
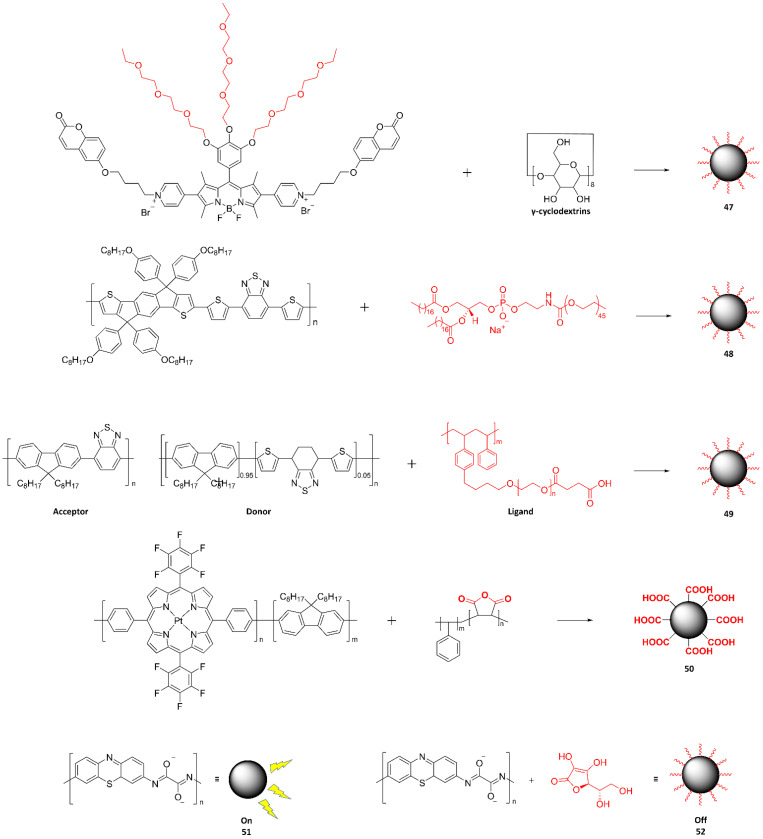
Chemical structures of the polymer probes constituting polymer dots when over two molecules are used.

**Figure 17:**
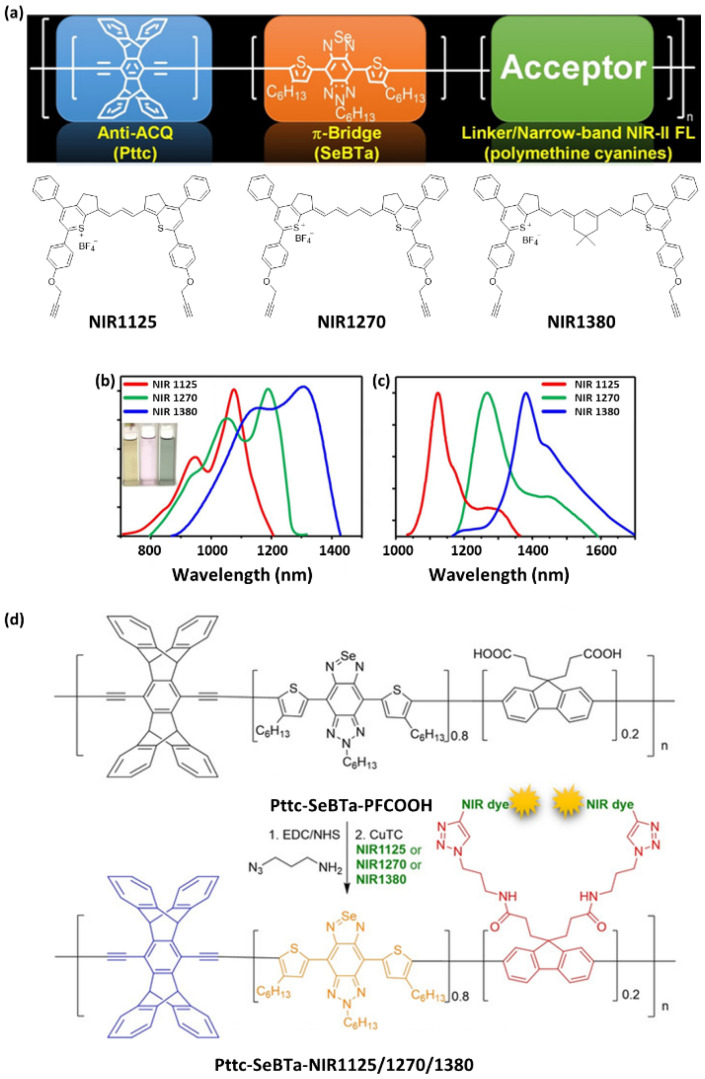
(**a**) Chemical structures of polymethine dye-conjugated semiconducting polymers. Three types of NIR-II fluorescent polymethine dyes (NIR1125, NIR1270, and NIR1380) were synthesized with activable alkyne functional groups for further conjugation with the semiconducting polymer. (**b**) Absorption spectra of NIR1125 (red line), NIR1270 (green line), and NIR1380 (blue line) in CH_2_Cl_2_. The middle-left inset shows a photograph of the dye solutions. (**c**) Emission spectra of NIR1125 (red line), NIR1270 (green line), and NIR1380 (blue line) in CH_2_Cl_2_. (**d**) Synthesis of a semiconducting polymer bearing carboxylic acid groups for further transformation to azide groups, followed by CuAAC click reactions with polymethine dyes. Reprinted with permission from Ref. [8]. Copyright 2020, John Wiley and Sons.

Figure 14, Figure 15 and Figure 16 summarize the molecular structures of photoluminescent Pdots that have been employed for bioimaging in recent studies. Figure 14 and Figure 15 present single-polymer probes, and Figure 16 shows examples of mixing two or more molecules at an appropriate ratio to prepare multi-component polymer probes. Table 5 summarizes the excitation peak, emission peak, and Stokes shifts of reported polymer probes. To date, there have been only limited examples of acquiring polymer probes with both absorption and emission maxima in the NIR-II window. Figure 17 presents a good example of the design of polymer probes to obtain desirable photophysical properties. NIR-II probes **24**–**26** are semiconducting polymers with a bulky architecture to provide anti-ACQ properties [8]. Both the rigid three-dimensional Pttc segment and the bulky SeBTa segment may provide steric hindrance to prevent the ACQ phenomenon of post-linked NIR-II fluorene-based monomers (fluorene derivatives with alkyne-functionalized polymethine-cyanine dyes). The covalent binding of the polymers with the NIR-II fluorophores is an efficient strategy to circumvent the fluorophore diffusion/leakage encountered by small-molecule fluorophore-entrapped/encapsulated polymeric nanoparticles. It is important to note that the fluorophore leaching problem may cause inconsistent optical signals and potential cytotoxicity, particularly for in vivo imaging applications. The Pttc and polyfluorene segments resulted in an absorption peak at ~450 nm and the SeBTa segment had an absorption peak at ~820 nm, while the polymethine-cyanine monomers were responsible for broad absorption ranging from 900 to 1400 nm. This indicates that probes **24**–**26** can be readily excited by long-wavelength laser sources depending on the measurement condition required for specific applications.

As another example, Habuchi and colleagues [49] synthesized probes **27**–**36** using a conjugated polymer with a bent and twisted structure to suppress the generation of fluorescence quenching. Specifically, probes **27**–**36** comprised an NIR-emitting polycarbazole-based donor-acceptor-type conjugated polymer and a polyfluorene-based planar conjugated polymer. The fluorescence brightness of the polymer probes was found to depend on (i) fluorescence quenching inside the polymer chains and (ii) intramolecular twisting between the donor and acceptor moieties of the polymer. These findings provide important insight, at the molecular level, into the development of NIR polymer probes with a high quantum yield. 

### 4.2. Nanoparticulate Polymer Probes

**Figure 18:**
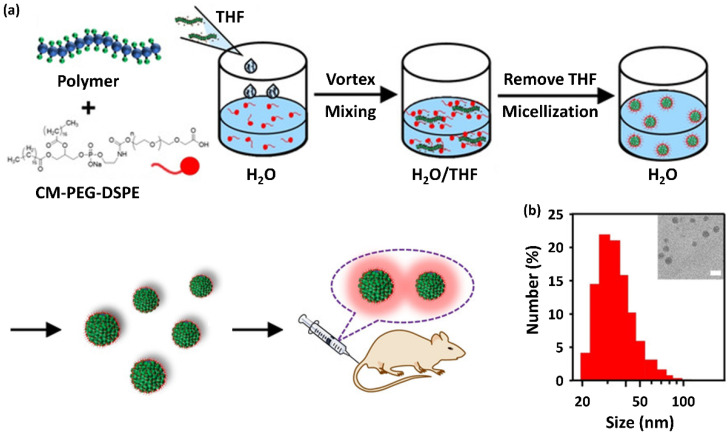
(**a**) Schematic showing the preparation of lipid-protected polymer dots (Pdots) for in vivo bioimaging. (**b**) Representative hydrodynamic sizes of probe **24** Pdots measured by dynamic light scattering. The upper-right inset represents the corresponding transmission electron microscopy image with a scale bar of 50 nm. Reprinted with permission from Ref. [8]. Copyright 2020, John Wiley and Sons.

To date, the most common method for preparing nanoparticulate polymer probes (mostly referred to as Pdots) has been the nanoprecipitation method. The preparation of NIR-II-probe **24**–**26**-based Pdots with surface carboxyl groups, illustrated in Figure 18a, followed the traditional nanoprecipitation method by co-precipitating amphiphilic lipids into the Pdots. The probe **25** Pdots were optically unstable in aqueous solutions, especially under light excitation, whereas they were stable in most organic solvents. By contrast, probe **24** and **26** Pdots were highly stable in organic solvents. The resulting Pdots showed hydrodynamic diameters of 35–73 nm. Figure 18b shows the particle size distribution of probe **31** Pdots along with their corresponding TEM images.

**Figure 19:**
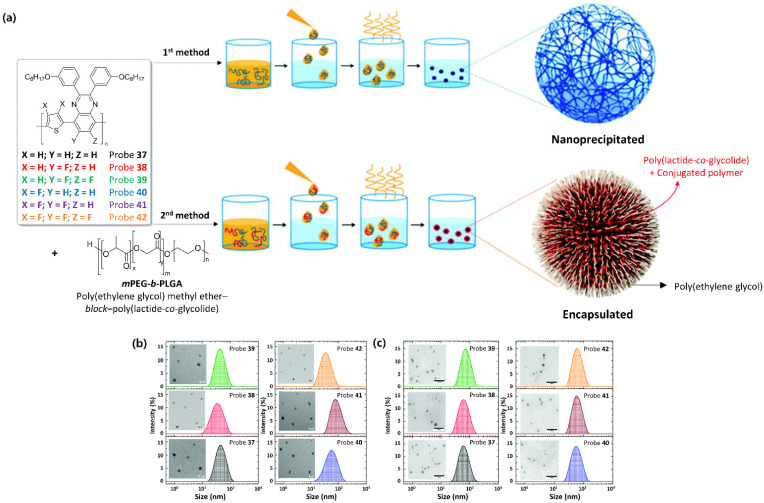
(**a**) Schematic representation of the conjugated polymer nanoparticles prepared through nanoprecipitation (top) and encapsulation (bottom). (**b**,**c**) Photographs that confirm the morphology by manufacturing polymer dots (Pdots) with a photoluminescence polymer. (**b**) Size distribution of aqueous probe **37**–**42** nanoparticles prepared via the nanoprecipitation method determined by dynamic light scattering (DLS) measurements and their morphology determined using transmission electron microscopy (TEM). (**c**) Size distribution of aqueous probe **37**–**42** Pdots prepared via the encapsulation method determined by DLS measurements and their morphology determined using TEM. Reprinted with permission from Ref. [121]. Copyright 2020, Royal Society of Chemistry.

The size uniformity of Pdots is also an important issue for bioimaging applications [253,254,255,256]. Recently, a few studies have confirmed the successful preparation of uniform-sized Pdots. Chochos and colleagues [121] reported the preparation of NIR Pdots, consisting of probes **37**–**42**, via both nanoprecipitation and encapsulation methods, as illustrated in Figure 19. For nanoprecipitation, each polymer was dissolved in THF, which is an aprotic polar solvent with a low boiling point, but is miscible with water. Subsequently, the polymer THF solution was added dropwise into water under sonication. The THF is readily evaporated even at room temperature, resulting in solidified nanoparticles. However, for encapsulation, the polymer was dissolved with an amphiphilic block copolymer, poly(ethylene glycol)methyl ether-block-poly(lactide-*co*-glycolide) (*m*PEG-*b*-PLGA), in THF. Note that *m*PEG-*b*-PLGA is an FDA-approved and metabolizable polymer. The rest of the procedure was the same as that described for the nanoprecipitation method. Stable aqueous-phase Pdots were then obtained through aggregate formation via hydrophobic interactions between the polymer chains. In the case of encapsulation, the hydrophobic PLGA chains would be liable to entangle with the polymer chains, while the hydrophilic PEG chains would extend into the aqueous phase. The obtained conjugated polymer nanoparticles (CPNs) were then filtered through a 0.2 μm cellulose acetate filter. The resulting concentrations (before filtration) of CPNs prepared via the nanoprecipitation method were 2.8 ppm for the TQ polymer and 4.3 ppm for all the others, but it was 143 ppm for CPNs prepared via the encapsulation method. TEM observation revealed a distinguishable, approximately spherical shape for all nanoparticles (Figure 19b). The diameters of the nanoparticles ranged from 24 nm to 40 nm for the nanoprecipitation method and from 17 nm to 20 nm for the encapsulation method. These average particle sizes, less than 50 nm, indicate the availability of Pdots for various biological applications [257,258,259]. 

**Figure 20:**
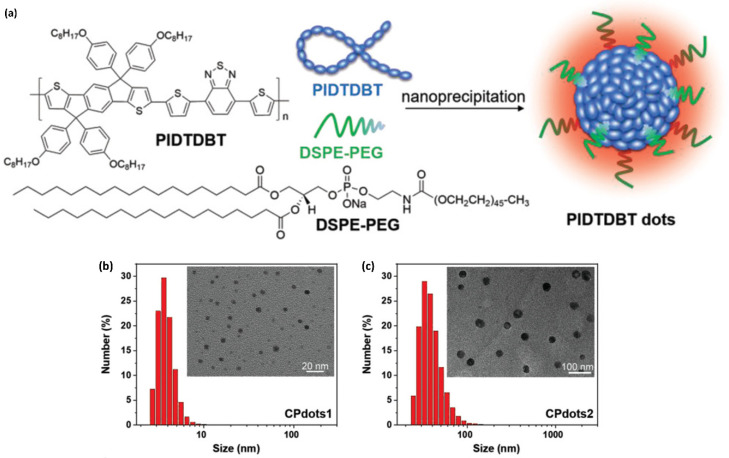
(**a**) Chemical structures of probe **48** and DSPE-PEG polymers and a schematic illustration for probe **48** Pdots synthesis. Hydrodynamic size distributions of DSPE-PEG:probe **48** weight ratios of (**b**) 10:1 and (**c**) 2:1 in aqueous media. The insets are the corresponding TEM images of 10:1 and 2:1. Reprinted with permission from Ref. [251]. Copyright 2019, Wiley-VCH Verlag GmBH & Co. KGAA.

As shown in Figure 20, probe **48** was designed to include strong electron-donating and electron-deficient units into the polymer backbone, which resulted in strong ICT emission at long wavelengths. The π-stacking between the polymer backbones was minimized by adding bulky 4-(octyloxy)phenyl groups, which would prevent undesirable intermolecular aggregation to yield highly efficient fluorescence inside the nanoparticles. Probe **48** was only soluble in organic solvents such as THF and toluene. Therefore, for biological applications, probe **48** was formulated into Pdots with the aid of an amphiphilic copolymer, 1,2-distearoyl-sn-glycero-3-phosphoethanolamine-*N*-[methoxy(polyethylene glycol)-2000] (DSPE-PEG), using a nanoprecipitation method (Figure 20a). Typically, a THF solution containing dissolved probe **48** and DSPE-PEG was quickly injected into water, and then this mixture solution was subject to continuous probe sonication. The hydrophobic polymer would aggregate into small nanoparticles with the hydrophobic segment of DSPE-PEG in aqueous solution, while the hydrophilic PEG chains would extend to the aqueous phase. Importantly, the hydrodynamic size of the resulting Pdots depended on the amount of DSPE-PEG used. The hydrodynamic sizes of the Pdots were 4 nm (probe **48** Pdot1) and 34 nm (probe **48** Pdot2) at DSPE-PEG-to-probe **48** weight ratios of 10:1 and 2:1 ratio, respectively (Figure 20b,c). The zeta potentials of the Pdots were measured to be approximately −45 to −51 mV, indicating that they have good colloidal stability in the aqueous phase.

### 4.3. In Vivo Bioimaging

**Figure 21:**
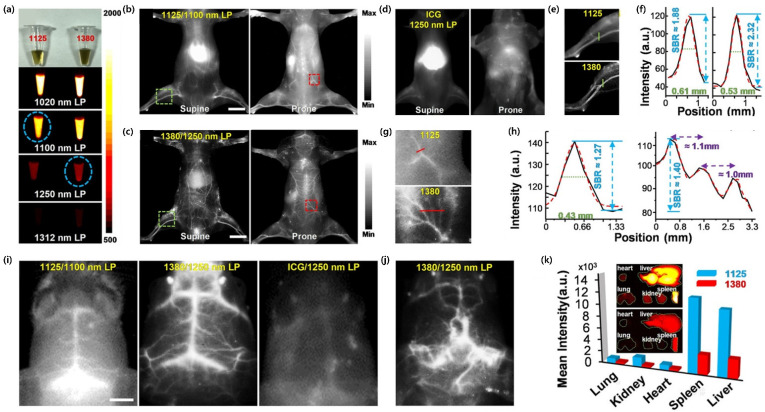
(**a**) Photographs of probe **24** Pdots (**left**) and probe **26** Pdots (**right**) under ambient light (first panel) and under 808 nm laser excitation with a 1020 nm LP filter (second panel), 1100 nm LP filter (third panel), 1250 nm LP filter (fourth panel), and 1312 nm LP filter (fifth panel). (**b**) Whole-body imaging of live mice intravenously injected with probe **24** Pdots in supine (**left**) and prone (**right**) positions with a 1100 nm LP filter. (**c**) Whole-body imaging of live mice intravenously injected with probe **26** Pdots in supine (**left**) and prone (**right**) positions with a 1250 nm LP filter. (**d**) Whole-body imaging of live mice intravenously injected with ICG in supine (**left**) and prone (**right**) positions with a 1250 nm LP filter. (**e**) Enlarged view of the mouse hindlimb vasculature in the green squares of (**b**) (**upper** panel) and (**c**) (**bottom** panel), respectively. (**f**) Cross-sectional intensity profiles along the green lines in (**e**) for probe **24** Pdots (**left**) and probe **26** Pdots (**right**), respectively. (**g**) Enlarged view of the area close to the spinal cord in the red squares of (**b**) (**upper** panel) and (**c**) (**bottom** panel), respectively. (**h**) Cross-sectional intensity profiles along the red lines in (**g**) for probe **24** Pdots (**left**) and probe **26** Pdots (right), respectively. (**i**) NIR-II fluorescence imaging of the wild-type C57BL/6 mouse brain vasculature after intravenous injection of probe **24** Pdots (**left**, 1100 nm LP filter), probe **26** Pdots (**middle**, 1250 nm LP filter), and ICG (**right**, 1250 nm LP filter). (**j**) NIR-II fluorescence imaging of the ND2:SmoA1 mouse brain vasculature after intravenous injection of probe **26** Pdots (1250 nm LP filter). (**k**) Accumulation of probe **24** Pdots (**upper** panel in the inset) and probe **26** Pdots (**bottom** panel in the inset) in the major excised organs at 24 h post-injection. Their corresponding quantitative mean fluorescence intensities are also plotted. The scale bars are 10 mm, 10 mm, and 5 mm in (**b**), (**c**), and (**i**), respectively. Reprinted with permission from Ref. [8]. Copyright 2020, John Wiley and Sons.

As one of the most recent studies on Pdots-based in vivo bioimaging, Chan and colleagues [8] reported the synthesis of probe **24** and probe **26** Pdots for in vivo non-invasive fluorescence imaging in mice. Prior to in vivo applications, they performed MTT assays to evaluate the cytotoxicity of Pdots at different concentrations, which confirmed that Pdots have only minimal cell toxicity. As shown in Figure 21a, irradiation was performed with an 808 nm laser source and low excitation power of 5 mW cm^−2^ to minimize photodamage to mice as well as the Pdots, which was equipped with different LP filters (1020, 1100, 1250, and 1312 nm). The use of a shorter LP filter (i.e., 1020 nm) was advantageous to achieve stronger fluorescence. However, the spatial resolution and the SBR for in vivo imaging showed the opposite trend due to strong background interference. By considering this result, a 1100 nm LP filter and a 1250 nm LP filter were used for probe **24** Pdots (traditional NIR-II) and probe **26** Pdots (NIR-IIb), respectively. 

Whole-body imaging in live mice was then performed by intravenously injecting the two Pdots separately into mice via the tail vein and comparing their performance (Figure 21b–e). The excitation power laser density was 5 mW cm^−2^ with an exposure time of 800 ms. The mice were intravenously injected through the tail vein with 200 μL of Pdots (1.6 mg mL^−1^) or ICG (200 μg mL^−1^). NIR-IIb imaging was performed at 10 min post-injection. For ICG control, all experimental conditions, including the laser power and filter sets, were the same as those used in the experiments with probe **26** Pdots, except that the acquisition time was 5 min post-injection due to the fast renal clearance rate of ICG. Fluorescence imaging performed by using probe **26** Pdots equipped with a 1250 nm LP filter revealed a significantly higher SBR than found with the 1125 nm LP filter (Figure 21b,c). By contrast, the imaging resolution obtained by ICG appeared to be very poor, as presented in Figure 21d. The two Pdots showed a similar spatial resolution of 0.53–0.61 mm (Figure 21e), whereas the SBR of probe **26** Pdots (SBR ~2.3) was approximately 1.2-times higher than that of probe **24** Pdots (Figure 21f). Importantly, the above results demonstrated the advantages of NIR-IIb imaging with nearly zero autofluorescence and minimal photon scattering. In addition, as shown in Figure 21g,h, the blood vessels close to the spinal cord were also readily visualized, demonstrating that probe **26** Pdots rendered a higher resolution due to low background interference. The cerebral blood vessels of the mouse through the intact scalp and skull were distinctly visualized along with the delicate vascular structures (Figure 21i). The high resolution of NIR-IIb imaging was again highlighted when using probe **26** Pdots (middle panel in Figure 21i). The purpose of this study was to diagnose the malignant brain tumor in vivo among the vascular morphology in the mouse brain, using the ND2:SmoA1 transgenic mouse as the model. As shown in Figure 21j, the brain vasculature in the ND2:SmoA1 mouse exhibited a major area of structural disorder in the brain vasculature. By contrast, the brain vasculature in the wild-type C57BL/6 mouse showed an orderly arrangement and distribution in the brain. The mice were further anatomized to analyze the distribution of the Pdots in major organs, where fluorescence was found mostly in the liver, kidney, and spleen (Figure 21k). The high accumulation of Pdots in the liver and spleen indicated that the hepatobiliary clearance system was the main metabolic pathway of Pdots. These results demonstrated that Pdots are suitable for deep-tissue NIR-II imaging, enabling the early diagnosis of diseases associated with a brain blood vessel abnormality.

**Figure 22:**
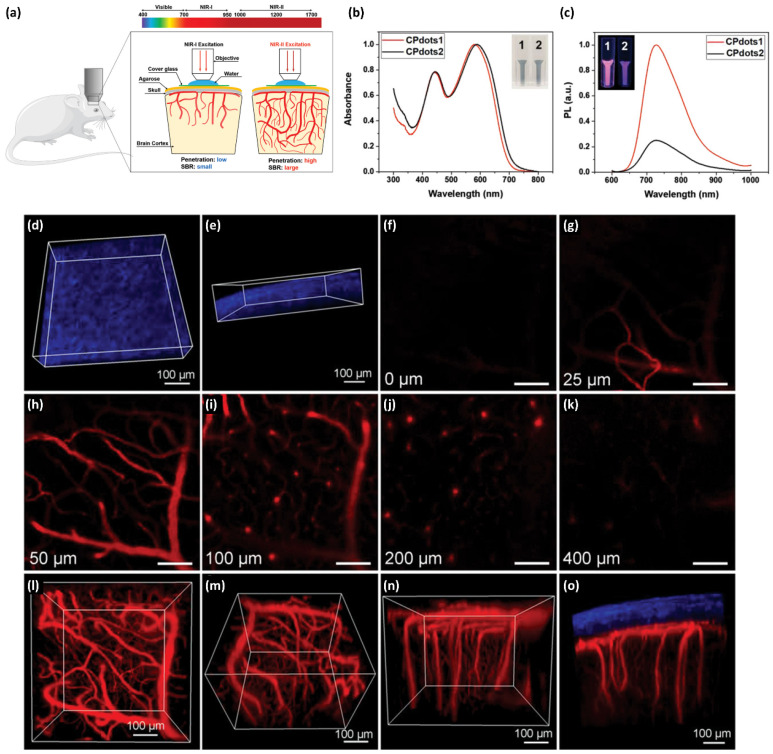
(**a**) Schematic illustration of NIR-I and NIR-II excited with in vivo 2PF imaging of the mouse brain. (**b**) Normalized UV–vis absorption spectra of probe **48** Pdots in aqueous media; inset is the digital photograph of the two probe **48** Pdots in cuvettes. (**c**) Photoluminescence spectra of probe **48** Pdots at a concentration of 10 µg mL^−1^ in aqueous solution; the inset shows a digital photograph of the two probe **48** Pdots in cuvettes under a UV lamp. In vivo 2PF imaging of probe **48** Pdot-stained mouse brain blood vessels with an intact skull. (**d**,**e**) Three-dimensional reconstructed second harmonic generation (SHG) images of the mouse skull. (**f**–**k**) 2PF images of probe **48** Pdot-stained brain blood vessels at various vertical depths. (**l**–**n**) Three-dimensional reconstructed 2PF images of the brain blood vessels. (**o**) Three-dimensional reconstructed image showing the position of the skull and the brain blood vessels network below the skull. Excitation for SHG: 950 nm; excitation for 2PF: 1200 nm. Emissions were collected within 455–500 nm for SHG and 660–750 nm for CPdots1. Each frame was acquired in 3.22 s. The length of scale bars in all images is 100 µm. Reprinted with permission from Ref. [251]. Copyright 2019, John Wiley and Sons.

Another non-invasive brain imaging method was also developed using probe **48** Pdots synthesized by the Liu group [251] (Figure 22). Two-photon fluorescence (2PF) microscopy with NIR light excitation was used as a non-invasive tool to examine the brain with a high spatial resolution and large imaging depth. Figure 22a demonstrates the deep in vivo 2PF imaging of the intact mouse brain using probe **48** Pdots, which are excitable by NIR-II light. Two Pdots of different size, namely probe **48** Pdot1 (~4 nm) and probe **48** Pdot2 (~34 nm), were prepared using the method described in Figure 20. The absorption and emission spectra of the probe **48** Pdots in aqueous solution are plotted in Figure 22b,c. The suspensions of probe **48** Pdot1 and probe **48** Pdot2 had similar absorption spectra with two peaks centered at approximately 445 nm and 600 nm. The photoluminescence spectra of both probe **48** Pdots exhibited a broad emission from 650 nm to 1000 nm. However, the fluorescence intensity of probe **48** Pdot1 was 4-times higher than that of probe **48** Pdot2 in aqueous media at the same concentration. The 2PF microscopy observation of **48** Pdots-labeled blood vessels in the same mouse brain with 800, 1040, and 1200 nm excitation indicated that longer-wavelength excitation reduced the background noise and improved the SBR of images. In a mouse model with a cranial window, in vivo 2PF imaging of probe **48** Pdots-stained brain blood vessels was achieved with an SBR of ~6 at a depth of 1010 µm for under 1200 nm femtosecond laser excitation. Through-skull 2PFM effectively visualized the blood vasculature architectures in the bone marrow within and beneath the skull. Importantly, a three-dimensional reconstruction of 2PF images of the brain vasculature network with high contrast and a large vertical depth of 400 µm through the intact skull was also successfully obtained (Figure 22d–o).

**Figure 23:**
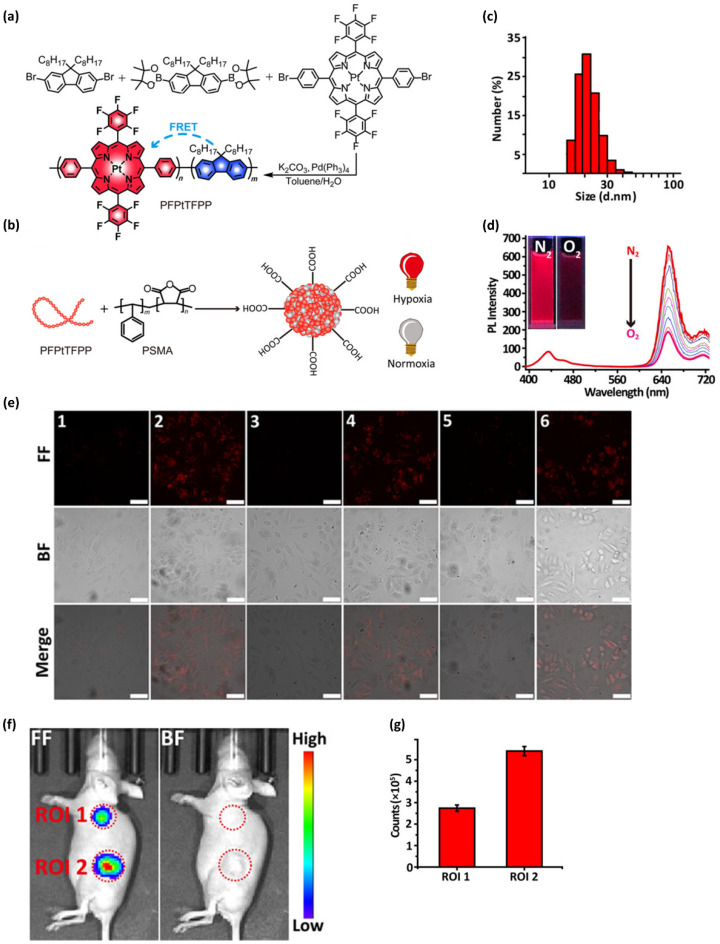
(**a**) Synthetic routes of polymer PFPtTFPP and (**b**) preparation of corresponding probe **50** Pdots. (**c**) Size distribution of probe **50** Pdots in aqueous solution measured by dynamic light scattering (DLS). (**d**) Emission spectra of probe **50** Pdots under different dissolved oxygen. Inset photographs show the oxygen and nitrogen atmosphere under 365 nm illumination. (**e**) Fluorescence imaging of MCF-7 cells without (1, 3, 5) and with (2, 4, 6) a cover glass. The top to bottom show fluorescence images, bright-field images, and combined bright-field and fluorescence images. The scale bar represents 75 μm. (**f**) Whole-body imaging of mice after subcutaneous (ROI 1) and intratumoral (ROI 2) injection of 1.6 mg mL^−1^ probe **50** Pdots solution. (**g**) Semiquantitative analysis of brightness in ROI 1 and ROI 2 areas. Reprinted with permission from Ref. [120]. Copyright 2019, Wiley-VCH Verlag GmBH & Co. KGAA.

The Wu group [120] reported the development of probe **50** Pdots for non-invasive in vivo hypoxia fluorescence imaging in mice. Probe **50** consisted of a hydrophobic polymer (PFPtTFPP) as an oxygen sensor with fluorene as a donor and Pt(II) porphyrin decorated with fluorobenzene as an acceptor (Figure 23a). As represented in Figure 23b,c, probe **50** Pdots with a size of approximately 21 nm were prepared using nanoprecipitation with poly(styrene-*co*-maleic anhydride). Blue and red emissions were observed from the Pdots under 374 nm excitation in an O_2_ atmosphere due to the fluorene moieties and Pt(II) porphyrin, respectively (Figure 23d). The capability of probe 50 Pdots for hypoxia imaging was first evaluated in vitro with MCF-7 cancer cells (Figure 23e). Initially, almost no phosphorescence was detected under a 5% CO_2_ in air atmosphere. Subsequently, a cover glass with 2 mm thickness and 10 mm diameter was placed on the cells for 1 h; the cover glass decreased the oxygen supply, thus generating hypoxia at the center of the glass. Fluorescence microscopy demonstrated that phosphorescence intensity significantly increased. After the cover glass was removed and the cells were incubated for another 10 min under 5 % CO_2_ in air, the phosphorescence intensity recovered to its initial weak state. Cycles of hypoxia–normoxia produced by repeatedly placing and removing the cover glass resulted in the same phosphorescence intensity changes over another two cycles. These results demonstrated that the probe **50** Pdots can present high reversibility for detecting repeated hypoxia–normoxia cycles. Next, hypoxia imaging in vivo was performed with subcutaneously tumor-implanted nude mice. The mice were intratumorally and subcutaneously injected with a probe **50** Pdots solution (10 μL, 1.6 mg mL^−^^1^), respectively. Whole-body imaging showed that the fluorescence intensity in the tumor area (region of interest (ROI) 2) was much higher than that of the subcutaneous injection area (ROI 1) (Figure 23f,g). This is mainly because the oxygen concentration in the tumor area is close to 0% and, thus, lower than that in the subcutaneous area. Collectively, these results indicated that probe **50** Pdots are highly sensitive to oxygen and, thus, have strong potential for hypoxia imaging in vivo.

## 5. Conclusions

To date, various efforts have been devoted to developing novel bioimaging techniques for practical applications. Photoluminescent probes such as small molecules, Pdots, CDs, graphene dots, MOFs, and COFs are promising candidates due to their interesting properties. This review illustrates the recent advances in the bioimaging strategies using photoluminescent small molecules and Pdots for in vivo applications. There is no doubt that these probes show excellent photoluminescent performance that can be utilized for in vitro and in vivo imaging applications. We have highlighted some of the recently explored methods and strategies for the preparation, evaluation, and application of nanoparticulate photoluminescent probes. Small-molecule probes for bioimaging have shown limitations owing to their low water dispersion (dissolution) and lack of photostability. Recent studies using small molecules for bioimaging have tried to overcome these drawbacks by shaping them into nanoparticles, frequently with appropriate biocompatible amphiphiles. Moreover, the use of polymers has some common advantages such as good biocompatibility, low toxicity, a tunable structure for the biotarget, and ease of designing the NIR emission-range structure for enhancing their penetration to detect biomarkers. Pdots have further advantages of good photostability, good water dispersion, and easy functionalization. 

In summary, this review has highlighted cases in which recently published organic molecular probes, including small molecules and polymers, were successfully used for bioimaging, along with the strategies for designing their structures, their strengths and weaknesses, and actual bioimaging effects. The importance of the bioimaging field continues to grow annually. In particular, due to global pandemics such as COVID-19, the development of bioimaging technology is increasingly necessary to easily detect and diagnose diseases, which will lead to the emergence of new types of bioimaging fields. 

We hope that this review will help in the design of probes for future bioimaging research.

## Figures and Tables

**Table 1:** Summary of the probes currently used in bioimaging.

Probe Type	Advantage	Disadvantage	Strategy	Reference
Small molecule	- Well-defined chemical structure - Structural tunability - Low cytotoxicity - High cell permeability - Rapid metabolism - Various routes to structure modification and functionalization	- Lack of photostability - Photobleaching - Low molecular brightness (i.e., low extinction coefficient)	Designing and synthesizing molecular probes with absorbance/emission in near-infrared (NIR) regions for - Endowing deeper penetration - Improved spatial resolution - Lower non-target autofluorescence	[47,103,104,105,106,107,108,109,110,111,112,113,114,115,116]
Polymer dots	- Biocompatibility - Photostability - Excellent brightness - Fast radiative rate - Large absorption coefficient - Good water dispersibility	- Poor solubility/dispersion - Low light penetration depth	- Functionalizing the surface with appropriate chemical groups for enhanced solubility or further conjugation with other functional species - Tuning the emission wavelength to situate in the tissue transparency window	[8,14,49,117,118,119,120,121,122,123,124]
Carbon-based quantum dots (QDs): graphene QDs and carbon dots	- Eco-friendly, facile synthesis (carbon dots) - Excellent photostability - Biocompatibility - Low cytotoxicity	- Low quantum yield - Intrinsically hydrophobic surface	- Choosing appropriate precursors or post-synthetic doping to gain desirable surface and photophysical properties - Energy-efficient method: microwave and hydrothermal method	[8,21,22,23,125,126,127,128,129,130,131,132,133,134,135,136,137]
Metal organic framework/covalent organic frame-works	- High porosity/large surface - Tunable chemical structure - High color purity - Long lifetime - Tunable chemical composition - Possible modification post-synthesis - Available for diverse fields	- Difficult to control the particle size and shape for in vivo application - Little information on biological activity	- Encapsulating guest species into the interior pore - Post-synthetic modification to improve water solubility and to control the photophysical properties	[138,139,140,141,142,143,144,145,146,147,148,149,150,151,152,153,154]

**Table 2:** Summary of the chemical units of small molecule-based photoluminescence probes ^a^.

Full Name (Abbreviation)	Chemical Structure	Characteristic	Application	Ref.
Tetraphenylethylene (TPE)	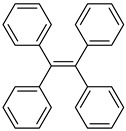	- Capability of self-organization - Conveniently incorporated into larger multicomponent assemblies with fluorophores	- OLEDs ^b^ - Chemo-imaging - Bioimaging	[194,195,196,197]
Benzobisthiadiazole (BBTD)	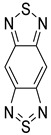	- Strong electron acceptor - Strong intramolecular charge transfer effect - Sensitive to harsh synthetic procedures	- Organic field-effect transistors - Photovoltaics - OLEDs	[198,199,200,201,202]
Triphenylamine (TPA)	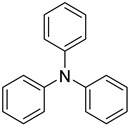	- Good electron donor - Carries a lone pair of electrons - Electroluminescence	- Organic optoelectronics - OLEDs - Photovoltaics	[203,204,205]
Diketopyrrolopyrrole (DPP)	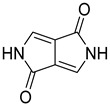	- Planar electron acceptor - Good photostability/thermal stability - High quantum yield - Wide absorption spectrum - High molar extinction coefficient - Strong electron-withdrawing ability - Large Stokes shift - Easy modification	- Fluorescent sensing - Photovoltaics - Bioimaging	[206,207,208,209,210,211]
Indocyanine green dye (ICG)	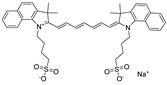	- Nontoxic - Near-infrared region of the absorption/fluorescence spectrum - Wide fluorescence spectrum - Reabsorption of itself due to overlap of absorption/fluorescence	- Bioimaging (e.g., determining cardiac output, hepatic function, liver and gastric blood flow, and for ophthalmic angiography)	[212,213,214]
Thiophene	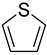	- Numerous electrons - Good electron donor - A basic building block of organic semiconductor materials - High photovoltaic performance	- Building block for organic conductors - Photovoltaics	[177,178,183,215,216,217,218]
Indeno[1,2-b]thiophene (IDT)	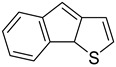	- Planar geometry - Strong electron donation - Can broaden the absorption wavelength range as well as enhance the charge transfer - Provides a large and unoccupied π-surface for effective intermolecular π−π interactions.	- Photocatalyst - OLEDs - Organic field-effect transistors - Photovoltaics	[219,220,221]
3,4-Ethylenedioxythiophene (EDOT)	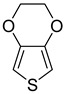	- Good electron donor - Mainly applied as a PEDOT ^c^ made through polymerization	- Building block for organic conductors - Transparent conductive coating - Electrode materials - OLEDs - Photovoltaics	[177,178,183,222]
Nitrobenzoxadiazole (NBD)	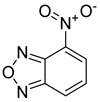	- Quite inexpensive - Easily conjugated with large Stokes shift - Good quantum yields - Fluorescent donor	- Sensing applications - Bioimaging - Energy transfer	[199,223,224,225]
Cyclopropenium ion derivatives: tris(dialkylamino)-cyclopropenium (TDAC) cation	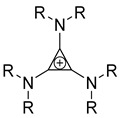	- Good electron donor - Outstanding stability - Good charge delocalization - Large Stokes shift	- Catalysts - Super bases - Ionic liquids - Polyelectrolytes - Photoelectronic redox-active chemistries	[226,227,228]
Triphenylphosphine (TPP)	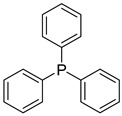	- Used as a phosphorus source - Air-robust - Cheap and good electron donor	- Catalyst for organic synthesis - Electrocatalytic oxidation - Water-splitting	[229,230,231]

^a^ Note that the toxicity of a unit cannot be directly associated with that of fluorescent probes based on the unit; ^b^ OLED: organic light-emitting diode; ^c^ PEDOT: poly(3,4-ethylenedioxythiophene).

**Table 3:** Summary of the optical properties with small molecule-based photoluminescence probes.

Material	λ_ex_ (nm)	λ_em_ (nm)	Stokes Shifts (nm)	HOMO ^a^ (eV)	LUMO ^b^ (eV)	Band Gap (eV)	Ref.
**1**	339	422	83	−0.27	−0.10	0.17	[105]
**2**	242	307	65	−0.30	−0.13	0.17	
**3**	274	304	30	−0.30	−0.17	0.12	
**4**	250	335	85	--	--	--	
**5**	249	338	89	−0.28	−0.17	0.11	
**6**	278	306	28	−0.28	−0.17	0.11	
**7**	274	306	32	−0.28	−0.17	0.11	
**8**	250	340	90	−0.28	−0.17	0.11	
**9**	337	421	84	−0.28	−0.20	0.08	
**10**	248	428	180	−0.28	−0.20	0.08	
**11**	248	431	183	−0.28	−0.20	0.08	
**12**	482	536	54	--	--	--	[103]
**13**	488	526	38	--	--	--	
**14**	524	555	31	--	--	--	
**15**	730 730 (NPs ^c^)	-- 898 (NPs)	-- 168 (NPs)	--	--	--	[111]
**16**	808 730 (NPs)	-- --	-- --	--	--	--	
**17**	610	665	55	--	--	--	[116]
**18**	700	900	200	--	--	1.91	
**19**	700	900	200	--	--	1.82	
**20**	643	922	279	−5.45	−3.67	1.78	[47]
**21**	762	1062	300	−4.84	−3.39	1.45	
**22**	725	1050	325	−4.83	−3.35	1.48	
**23**	805	1034	229	−4.50	−3.35	1.15	[112]

**^a^** HOMO: highest occupied molecular orbital; **^b^** LUMO: lowest unoccupied molecular orbital; **^c^** NPs: nanoparticles.

**Table 4:** Summary of the chemical units of polymer-based photoluminescence probes ^a^.

Full Name (Abbreviation)	Chemical Structure	Characteristics	Application
2,1,3-Benzothiadiazole (BTD)	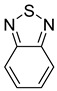	- Strong electron-withdrawing capacity - Candidate electron carriers - Highly polarized property - Induces intermolecular interactions	- Fungicides - Herbicides - Antibacterials - Plant growth and protection - Gene regulation
Pentiptycene	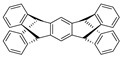	- Structural rigidity - Nonplanarity - Bulkiness - π-electron richness	- Microporous materials - Host–guest chemistry - Membranes
Triazole	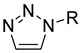	- Strong dipole moment - π-electron-deficient aromaticity - Hydrogen bond-accepting properties - Good metabolic stability	- Bioisosteres - Antibacterial/antifungal agents - Anticancer agents
Fluorene	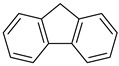	- Rigid planar structure - High photoluminescence efficiency - Good thermal stability - Low cytotoxicity	- OLEDs ^b^ - TADF ^c^ emitter
Spirobifluorene	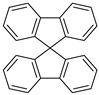	- Structural rigidity - Good thermal stability	- OLEDs - Organic photovoltaics
Quinoxaline	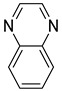	- Good charge-transporting properties - Dopant-free hole transporting materials - Various derivatives	- Dyes - Pharmaceuticals - Antibiotics
Boron-dipyrromethene (BODIPY)	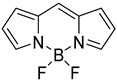	- High quantum yield - Photochemical and thermal robustness - High solubility in organic solvents - Ease of synthetic functionalization - High molar extinction coefficient and fluorescence quantum yield	- Sensors - Optoelectronic devices - Therapeutic application - Redox flow batteries
Porphyrin	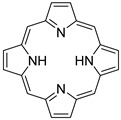	- Absorb light at wavelengths near 400 nm - Ability to coordinate with metal ions - Facile derivatization - Supramolecular self-assembly	- Light-harvesting applications - Biomimetic catalysis - Photodynamic therapy
Methylene blue	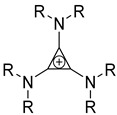	- High water solubility - Good electron donor - Good charge delocalization - Relatively low hydrogen bond donor capabilities	- Staining agent - Pharmaceutical agent - Polyelectrolytes - Nitrogen-based ligands - Photoelectronic redox-active chemistries

^a^ Note that the toxicity of a unit cannot be directly associated with that of fluorescent probes based on the unit; ^b^ OLED, organic light-emitting diode; ^c^ TADF: thermally activated delayed fluorescence.

**Table 5:** Summary of the optical properties of photoluminescence polymer dots (Pdots) used for bioimaging.

Material	Pdots λ_ex_ (nm)	Pdots λ_em_ (nm)	Stokes Shifts (nm)	Ref.
**24**	936, 1066 (Probe) 400, 820, 1082 (Pdots)	1125 (Probe) 1115 (Pdots)	59 (Probe) 33 (Pdots)	[8]
**25**	1050, 1188 (Probe) 400, 820, 1082 (Pdots)	1270 (Probe) 1225 (Pdots)	82 (Probe) 143 (Pdots)	
**26**	1140, 1270 (Probe) 400, 820, 1200 (Pdots)	1380 (Probe) 1300 (Pdots)	110 (Probe) 100 (Pdots)	
**27**	497	631	134	[49]
**28**	497	603	106	
**29**	501	659	158	
**30**	520	682	162	
**31**	593	653	60	
**32**	477, 560	710	150	
**33**	622	733	111	
**34**	835	1035	200	
**35**	736	1040	304	
**36**	812	934	122	
**37**	354, 600	668	68	[121]
**38**	338, 574	618	44	
**39**	319, 574	592	18	
**40**	538	600	62	
**41**	387, 498	571	73	
**42**	392, 456	554	98	
**43**	452	475	23	[118]
**44**	452	476	24	
**45**	449	474	25	
**46**	390	515	125	[119]
**47**	528	550	22	[122]
**48**	445, 600	725	125	[251]
**49**	470	645	175	[117]
**50**	--	651, 713	--	[120]
**51**	570	630	60	[252]
**52**	570	--	--	

## References

[1] Wang P., Fan Y., Lu L., Liu L., Fan L., Zhao M., Xie Y., Xu C., Zhang F. (2018). NIR-II nanoprobes in-vivo assembly to improve image-guided surgery for metastatic ovarian cancer. Nat. Commun..

[2] Qi J., Chen C., Zhang X., Hu X., Ji S., Kwok R.T.K., Lam J.W.Y., Ding D., Tang B.Z. (2018). Light-driven transformable optical agent with adaptive functions for boosting cancer surgery outcomes. Nat. Commun..

[3] Ceppi L., Bardhan N.M., Na Y., Siegel A., Rajan N., Fruscio R., Del Carmen M.G., Belcher A.M., Birrer M.J. (2019). Real-Time Single-Walled Carbon Nanotube-Based Fluorescence Imaging Improves Survival after Debulking Surgery in an Ovarian Cancer Model. ACS Nano.

[4] Jose D.A., Sakla R., Sharma N., Gadiyaram S., Kaushik R., Ghosh A. (2020). Sensing and Bioimaging of the Gaseous Signaling Molecule Hydrogen Sulfide by Near-Infrared Fluorescent Probes. ACS Sens..

[5] Li J.B., Liu H.W., Fu T., Wang R., Zhang X.B., Tan W. (2019). Recent Progress in Small-Molecule Near-IR Probes for Bioimaging. Trends Chem..

[6] Li K., Xu S., Xiong M., Huan S.Y., Yuan L., Zhang X.B. (2021). Molecular engineering of organic-based agents for in situ bioimaging and phototherapeutics. Chem. Soc. Rev..

[7] Christopherson C.J., Paisley N.R., Xiao Z., Algar W.R., Hudson Z.M. (2021). Red-Emissive Cell-Penetrating Polymer Dots Exhibiting Thermally Activated Delayed Fluorescence for Cellular Imaging. J. Am. Chem. Soc..

[8] Liu M.H., Zhang Z., Yang Y.C., Chan Y.H. (2021). Polymethine-Based Semiconducting Polymer Dots with Narrow-Band Emission and Absorption/Emission Maxima at NIR-II for Bioimaging. Angew. Chem. Int. Ed. Engl..

[9] Verma M., Chan Y.H., Saha S., Liu M.H. (2021). Recent Developments in Semiconducting Polymer Dots for Analytical Detection and NIR-II Fluorescence Imaging. ACS Appl. Bio Mater..

[10] Yoon H., Ahn J.-H., Barone P.W., Yum K., Sharma R., Boghossian A.A., Han J.-H., Strano M.S. (2011). Periplasmic Binding Proteins as Optical Modulators of Single-Walled Carbon Nanotube Fluorescence: Amplifying a Nanoscale Actuator. Angew. Chem. Int. Ed..

[11] Ahn J.-H., Kim J.-H., Reuel N.F., Barone P.W., Boghossian A.A., Zhang J., Yoon H., Chang A.C., Hilmer A.J., Strano M.S. (2011). Label-Free, Single Protein Detection on a Near-Infrared Fluorescent Single-Walled Carbon Nanotube/Protein Microarray Fabricated by Cell-Free Synthesis. Nano Lett..

[12] Danné N., Godin A.G., Gao Z., Varela J.A., Groc L., Lounis B., Cognet L. (2017). Comparative Analysis of Photoluminescence and Upconversion Emission from Individual Carbon Nanotubes for Bioimaging Applications. ACS Photonics.

[13] Huth K., Glaeske M., Achazi K., Gordeev G., Kumar S., Arenal R., Sharma S.K., Adeli M., Setaro A., Reich S. (2018). Fluorescent Polymer-Single-Walled Carbon Nanotube Complexes with Charged and Noncharged Dendronized Perylene Bisimides for Bioimaging Studies. Small.

[14] Nagai Y., Nakamura K., Yudasaka M., Shiraki T., Fujigaya T. (2020). Radical Polymer Grafting on the Surface of Single-Walled Carbon Nanotubes Enhances Photoluminescence in the Near-Infrared Region: Implications for Bioimaging and Biosensing. ACS Appl. Nano Mater..

[15] Le T.-H., Lee S., Heo E., Lee U., Lee H., Jo H., Yang K.S., Chang M., Yoon H. (2021). Controlled anisotropic growth of layered perovskite nanocrystals for enhanced optoelectronic properties. Chem. Eng. J..

[16] Le T.-H., Lee S., Jo H., Kim M., Lee J., Chang M., Yoon H. (2021). Deep Exciton Self-Trapping Cu-Based Perovskite Nanocrystals for Optoelectronic Applications. ACS Appl. Nano Mater..

[17] Le T.-H., Choi Y., Kim S., Lee U., Heo E., Lee H., Chae S., Im W.B., Yoon H. (2020). Highly Elastic and >200% Reversibly Stretchable Down-Conversion White Light-Emitting Diodes Based on Quantum Dot Gel Emitters. Adv. Opt. Mater..

[18] Le T.-H., Choi Y., Han H., Noh S., Park C.S., Kim S., Chae S., Kim H.J., Im W.B., Ha T.H. (2018). Highly Luminescent Quantum Dots in Remote-Type Liquid-Phase Color Converters for White Light-Emitting Diodes. Adv. Mater. Technol..

[19] Le T.-H., Lee S., Jo H., Jeong G., Chang M., Yoon H. (2021). Morphology-Dependent Ambient-Condition Growth of Perovskite Nanocrystals for Enhanced Stability in Photoconversion Device. J. Phys. Chem. Lett..

[20] Le T.-H., Kim S., Chae S., Choi Y., Park C.S., Heo E., Lee U., Kim H., Kwon O.S., Im W.B. (2020). Zero reduction luminescence of aqueous-phase alloy core/shell quantum dots via rapid ambient-condition ligand exchange. J. Colloid Interface Sci..

[21] Chung S., Revia R.A., Zhang M. (2021). Graphene Quantum Dots and Their Applications in Bioimaging, Biosensing, and Therapy. Adv. Mater..

[22] Lu H., Li W., Dong H., Wei M. (2019). Graphene Quantum Dots for Optical Bioimaging. Small.

[23] Younis M.R., He G., Lin J., Huang P. (2020). Recent Advances on Graphene Quantum Dots for Bioimaging Applications. Front. Chem..

[24] Yu Z., Eich C., Cruz L.J. (2020). Recent Advances in Rare-Earth-Doped Nanoparticles for NIR-II Imaging and Cancer Theranostics. Front. Chem..

[25] Zhang X., He S., Ding B., Qu C., Chen H., Sun Y., Zhang R., Lan X., Cheng Z. (2021). Synergistic strategy of rare-earth doped nanoparticles for NIR-II biomedical imaging. J. Mater. Chem. B.

[26] Zhu X., Zhang J., Liu J., Zhang Y. (2019). Recent Progress of Rare-Earth Doped Upconversion Nanoparticles: Synthesis, Optimization, and Applications. Adv. Sci..

[27] Cai Y., Tang C., Wei Z., Song C., Zou H., Zhang G., Ran J., Han W. (2021). Fused-Ring Small-Molecule-Based Bathochromic Nano-agents for Tumor NIR-II Fluorescence Imaging-Guided Photothermal/Photodynamic Therapy. ACS Appl. Bio Mater..

[28] Ding Y., Tong Z., Jin L., Ye B., Zhou J., Sun Z., Yang H., Hong L., Huang F., Wang W. (2022). An NIR Discrete Metallacycle Constructed from Perylene Bisimide and Tetraphenylethylene Fluorophores for Imaging-Guided Cancer Radio-Chemotherapy. Adv. Mater..

[29] Yang Z., Zhang Z., Lei Z., Wang D., Ma H., Tang B.Z. (2021). Precise Molecular Engineering of Small Organic Phototheranostic Agents toward Multimodal Imaging-Guided Synergistic Therapy. ACS Nano.

[30] Lee S., Kwon O.S., Lee C.-S., Won M., Ban H.S., Ra C.S. (2017). Synthesis and biological evaluation of kresoxim-methyl analogues as novel inhibitors of hypoxia-inducible factor (HIF)-1 accumulation in cancer cells. Bioorganic Med. Chem. Lett..

[31] Smith A.M., Mancini M.C., Nie S. (2009). Bioimaging: Second window for in vivo imaging. Nat. Nanotechnol..

[32] König K., Becker T.W., Fischer P., Riemann I., Halbhuber K.J. (1999). Pulse-length dependence of cellular response to intense near-infrared laser pulses in multiphoton microscopes. Opt. Lett..

[33] Umanzor-Alvarez J., Wade E.C., Gifford A., Nontapot K., Cruz-Reese A., Gotoh T., Sible J.C., Khodaparast G.A. (2011). Near-infrared laser delivery of nanoparticles to developing embryos: A study of efficacy and viability. Biotechnol. J..

[34] Chen Y.S., Zhao Y., Yoon S.J., Gambhir S.S., Emelianov S. (2019). Miniature gold nanorods for photoacoustic molecular imaging in the second near-infrared optical window. Nat. Nanotechnol..

[35] Duan X., Zhang G.Q., Ji S., Zhang Y., Li J., Ou H., Gao Z., Feng G., Ding D. (2022). Activatable Persistent Luminescence from Porphyrin Derivatives and Supramolecular Probes with Imaging-Modality Transformable Characteristics for Improved Biological Applications. Angew. Chem. Int. Ed. Engl..

[36] Zhao M., Li B., Zhang H., Zhang F. (2020). Activatable fluorescence sensors for in vivo bio-detection in the second near-infrared window. Chem. Sci.

[37] Huang S., Lin C.W., Qi J., Iyer A.M., He Y., Li Y., Bardhan N.M., Irvine D.J., Hammond P.T., Belcher A.M. (2021). Surface Plasmon-Enhanced Short-Wave Infrared Fluorescence for Detecting Sub-Millimeter-Sized Tumors. Adv. Mater..

[38] Lang W., Yuan C., Zhu L., Du S., Qian L., Ge J., Yao S.Q. (2020). Recent advances in construction of small molecule-based fluorophore-drug conjugates. J. Pharm Anal..

[39] Shen Q., Wang S., Yang N.-D., Zhang C., Wu Q., Yu C. (2020). Recent development of small-molecule organic fluorophores for multifunctional bioimaging in the second near-infrared window. J. Lumin..

[40] Li L., Dong X., Li J., Wei J. (2020). A short review on NIR-II organic small molecule dyes. Dye. Pigment..

[41] Ding F., Chen S., Zhang W., Tu Y., Sun Y. (2017). UPAR targeted molecular imaging of cancers with small molecule-based probes. Bioorg. Med. Chem..

[42] Yin J., Ma Y., Li G., Peng M., Lin W. (2020). A versatile small-molecule fluorescence scaffold: Carbazole derivatives for bioimaging. Coord. Chem. Rev..

[43] Wang F., Wan H., Ma Z., Zhong Y., Sun Q., Tian Y., Qu L., Du H., Zhang M., Li L. (2019). Light-sheet microscopy in the near-infrared II window. Nat. Methods.

[44] Park C.S., Ha T.H., Kim M., Raja N., Yun H.S., Sung M.J., Kwon O.S., Yoon H., Lee C.S. (2018). Fast and sensitive near-infrared fluorescent probes for ALP detection and 3d printed calcium phosphate scaffold imaging in vivo. Biosens. Bioelectron..

[45] Chen S., Zhang W., Jiang X., Guo Y., Sun P., Wang W., Fan Q., Huang W. (2021). Bright NIR-II Fluorescent Small-Molecule Nanoparticles with Reduced Intermolecular Interaction for Targeted In Vivo Inflammation Imaging. ACS Appl. Polym. Mater..

[46] Li D., Qu C., Liu Q., Wu Y., Hu X., Qian K., Chang B., He S., Yuan Y., Li Y. (2019). Monitoring the Real-Time Circulatory System-Related Physiological and Pathological Processes In Vivo Using a Multifunctional NIR-II Probe. Adv. Funct. Mater..

[47] Li Y., Liu Y., Li Q., Zeng X., Tian T., Zhou W., Cui Y., Wang X., Cheng X., Ding Q. (2020). Novel NIR-II organic fluorophores for bioimaging beyond 1550 nm. Chem. Sci..

[48] Ding F., Li C., Xu Y., Li J., Li H., Yang G., Sun Y. (2018). PEGylation Regulates Self-Assembled Small-Molecule Dye-Based Probes from Single Molecule to Nanoparticle Size for Multifunctional NIR-II Bioimaging. Adv. Healthc. Mater..

[49] Piwoński H., Li W., Wang Y., Michinobu T., Habuchi S. (2019). Improved Fluorescence and Brightness of Near-Infrared and Shortwave Infrared Emitting Polymer Dots for Bioimaging Applications. ACS Appl. Polym. Mater..

[50] Wu Y., Ruan H., Zhao R., Dong Z., Li W., Tang X., Yuan J., Fang X. (2018). Ultrastable Fluorescent Polymer Dots for Stimulated Emission Depletion Bioimaging. Adv. Opt. Mater..

[51] Frédéric L., Desmarchelier A., Favereau L., Pieters G. (2021). Designs and Applications of Circularly Polarized Thermally Activated Delayed Fluorescence Molecules. Adv. Funct. Mater..

[52] Peng C.C., Yang S.Y., Li H.C., Xie G.H., Cui L.S., Zou S.N., Poriel C., Jiang Z.Q., Liao L.S. (2020). Highly Efficient Thermally Activated Delayed Fluorescence via an Unconjugated Donor-Acceptor System Realizing EQE of Over 30. Adv. Mater..

[53] Bryden M.A., Zysman-Colman E. (2021). Organic thermally activated delayed fluorescence (TADF) compounds used in photocatalysis. Chem. Soc. Rev..

[54] Ikeda N., Oda S., Matsumoto R., Yoshioka M., Fukushima D., Yoshiura K., Yasuda N., Hatakeyama T. (2020). Solution-Processable Pure Green Thermally Activated Delayed Fluorescence Emitter Based on the Multiple Resonance Effect. Adv. Mater..

[55] Zhao J., Dong H., Yang H., Zheng Y. (2019). Solvent-Polarity-Dependent Excited-State Behavior and Thermally Active Delayed Fluorescence for Triquinolonobenzene. ACS Appl. Bio Mater..

[56] Yang Y.-C., Liu M.-H., Yang S.-M., Chan Y.-H. (2021). Bimodal Multiplexed Detection of Tumor Markers in Non-Small Cell Lung Cancer with Polymer Dot-Based Immunoassay. ACS Sens..

[57] Chen H., Yu J., Men X., Zhang J., Ding Z., Jiang Y., Wu C., Chiu D.T. (2021). Reversible Ratiometric NADH Sensing Using Semiconducting Polymer Dots. Angew. Chem. Int. Ed. Engl..

[58] Yuan X., Bai F., Ye H., Zhao H., Zhao L., Xiong Z. (2021). Smartphone-assisted ratiometric fluorescence sensing platform and logical device based on polydopamine nanoparticles and carbonized polymer dots for visual and point-of-care testing of glutathione. Anal. Chim. Acta.

[59] Huang L., Jin J., Ao L., Jiang C., Zhang Y., Wen H.M., Wang J., Wang H., Hu J. (2020). Hierarchical Plasmonic-Fluorescent Labels for Highly Sensitive Lateral Flow Immunoassay with Flexible Dual-Modal Switching. ACS Appl. Mater. Interfaces.

[60] Luo Y., Han Y., Hu X., Yin M., Wu C., Li Q., Chen N., Zhao Y. (2019). Live-cell imaging of octaarginine-modified polymer dots via single particle tracking. Cell Prolif..

[61] Paisley N.R., Halldorson S.V., Tran M.V., Gupta R., Kamal S., Algar W.R., Hudson Z.M. (2021). Near-Infrared-Emitting Boron-Difluoride-Curcuminoid-Based Polymers Exhibiting Thermally Activated Delayed Fluorescence as Biological Imaging Probes. Angew. Chem. Int. Ed. Engl..

[62] Wang G., Yang L., Li C., Yu H., He Z., Yang C., Sun J., Zhang P., Gu X., Tang B.Z. (2021). Novel strategy to prepare fluorescent polymeric nanoparticles based on aggregation-induced emission via precipitation polymerization for fluorescent lateral flow assay. Mater. Chem. Front..

[63] Mayder D.M., Tonge C.M., Nguyen G.D., Tran M.V., Tom G., Darwish G.H., Gupta R., Lix K., Kamal S., Algar W.R. (2021). Polymer Dots with Enhanced Photostability, Quantum Yield, and Two-Photon Cross-Section using Structurally Constrained Deep-Blue Fluorophores. J. Am. Chem. Soc..

[64] Wang Z., Wang X., Zhang Y., Xu W., Han X. (2021). Principles and Applications of Single Particle Tracking in Cell Research. Small.

[65] Park J., Bailey E.J., Composto R.J., Winey K.I. (2020). Single-Particle Tracking of Nonsticky and Sticky Nanoparticles in Polymer Melts. Macromolecules.

[66] Xu M., Ma C., Zhou J., Liu Y., Wu X., Luo S., Li W., Yu H., Wang Y., Chen Z. (2019). Assembling semiconductor quantum dots in hierarchical photonic cellulose nanocrystal films: Circularly polarized luminescent nanomaterials as optical coding labels. J. Mater. Chem. C.

[67] McHugh K.J., Jing L., Behrens A.M., Jayawardena S., Tang W., Gao M., Langer R., Jaklenec A. (2018). Biocompatible Semiconductor Quantum Dots as Cancer Imaging Agents. Adv. Mater..

[68] Pu Y., Cai F., Wang D., Wang J.-X., Chen J.-F. (2018). Colloidal Synthesis of Semiconductor Quantum Dots toward Large-Scale Production: A Review. Ind. Eng. Chem. Res..

[69] Xia M., Luo J., Chen C., Liu H., Tang J. (2019). Semiconductor Quantum Dots-Embedded Inorganic Glasses: Fabrication, Luminescent Properties, and Potential Applications. Adv. Opt. Mater..

[70] Xu X., Ray R., Gu Y., Ploehn H.J., Gearheart L., Raker K., Scrivens W.A. (2004). Electrophoretic Analysis and Purification of Fluorescent Single-Walled Carbon Nanotube Fragments. J. Am. Chem. Soc..

[71] Lee U., Heo E., Le T.-H., Lee H., Kim S., Lee S., Jo H., Yoon H. (2021). Carbon dots for epoxy curing: Anti-forgery patterns with long-term luminescent stability. Chem. Eng. J..

[72] Jiao Y., Gao Y., Meng Y., Lu W., Liu Y., Han H., Shuang S., Li L., Dong C. (2019). One-Step Synthesis of Label-Free Ratiometric Fluorescence Carbon Dots for the Detection of Silver Ions and Glutathione and Cellular Imaging Applications. ACS Appl. Mater. Interfaces.

[73] Jiang L., Ding H., Lu S., Geng T., Xiao G., Zou B., Bi H. (2020). Photoactivated Fluorescence Enhancement in F,N-Doped Carbon Dots with Piezochromic Behavior. Angew. Chem. Int. Ed. Engl..

[74] Zhu Z., Cheng R., Ling L., Li Q., Chen S. (2020). Rapid and Large-Scale Production of Multi-Fluorescence Carbon Dots by a Magnetic Hyperthermia Method. Angew. Chem. Int. Ed. Engl..

[75] Nguyen H.A., Srivastava I., Pan D., Gruebele M. (2020). Unraveling the Fluorescence Mechanism of Carbon Dots with Sub-Single-Particle Resolution. ACS Nano.

[76] Crista D.M.A., Esteves da Silva J.C.G., Pinto da Silva L. (2020). Evaluation of Different Bottom-up Routes for the Fabrication of Carbon Dots. Nanomaterials.

[77] Qu D., Sun Z. (2020). The formation mechanism and fluorophores of carbon dots synthesized via a bottom-up route. Mater. Chem. Front..

[78] Pillar-Little T.J., Wanninayake N., Nease L., Heidary D.K., Glazer E.C., Kim D.Y. (2018). Superior photodynamic effect of carbon quantum dots through both type I and type II pathways: Detailed comparison study of top-down-synthesized and bottom-up-synthesized carbon quantum dots. Carbon.

[79] Shi W., Han Q., Wu J., Ji C., Zhou Y., Li S., Gao L., Leblanc R.M., Peng Z. (2022). Synthesis Mechanisms, Structural Models, and Photothermal Therapy Applications of Top-Down Carbon Dots from Carbon Powder, Graphite, Graphene, and Carbon Nanotubes. Int. J. Mol. Sci..

[80] Novoselov K.S., Geim A.K., Morozov S.V., Jiang D., Zhang Y., Dubonos S.V., Grigorieva I.V., Firsov A.A. (2004). Electric Field Effect in Atomically Thin Carbon Films. Science.

[81] Chae S., Le T.-H., Park C.S., Choi Y., Kim S., Lee U., Heo E., Lee H., Kim Y.A., Kwon O.S. (2020). Anomalous restoration of sp2 hybridization in graphene functionalization. Nanoscale.

[82] Oh J.-S., Lee E.-J. (2021). Photodynamic Graphene Oxide Combined Alginate Hydrogel for Controlled Drug Release. Macromol. Res..

[83] Jin Y., Zheng Y., Podkolzin S.G., Lee W. (2020). Band gap of reduced graphene oxide tuned by controlling functional groups. J. Mater. Chem. C.

[84] Li G., Yoon K.Y., Zhong X., Wang J., Zhang R., Guest J.R., Wen J., Zhu X.Y., Dong G. (2018). A modular synthetic approach for band-gap engineering of armchair graphene nanoribbons. Nat. Commun..

[85] Dejpasand M.T., Saievar-Iranizad E., Bayat A., Montaghemi A., Ardekani S.R. (2020). Tuning HOMO and LUMO of three region (UV, Vis and IR) photoluminescent nitrogen doped graphene quantum dots for photodegradation of methylene blue. Mater. Res. Bull..

[86] Bafekry A., Neek-Amal M. (2020). Tuning the electronic properties of graphene–graphitic carbon nitride heterostructures and heterojunctions by using an electric field. Phys. Rev. B.

[87] Li G., Zhao S., Zhang Y., Tang Z. (2018). Metal-Organic Frameworks Encapsulating Active Nanoparticles as Emerging Composites for Catalysis: Recent Progress and Perspectives. Adv. Mater..

[88] Thomas-Hillman I., Laybourn A., Dodds C., Kingman S.W. (2018). Realising the environmental benefits of metal–organic frameworks: Recent advances in microwave synthesis. J. Mater. Chem. A.

[89] Xiao X., Zou L., Pang H., Xu Q. (2020). Synthesis of micro/nanoscaled metal-organic frameworks and their direct electrochemical applications. Chem. Soc. Rev..

[90] Müller-Buschbaum K., Beuerle F., Feldmann C. (2015). MOF based luminescence tuning and chemical/physical sensing. Microporous Mesoporous Mater..

[91] Côté A.P., Benin A.I., Ockwig N.W., O’Keeffe M., Matzger A.J., Yaghi O.M. (2005). Porous, Crystalline, Covalent Organic Frameworks. Science.

[92] Sun T., Xie J., Guo W., Li D.S., Zhang Q. (2020). Covalent–Organic Frameworks: Advanced Organic Electrode Materials for Rechargeable Batteries. Adv. Energy Mater..

[93] Faheem M., Aziz S., Jing X., Ma T., Du J., Sun F., Tian Y., Zhu G. (2019). Dual luminescent covalent organic frameworks for nitro-explosive detection. J. Mater. Chem. A.

[94] Yusran Y., Li H., Guan X., Li D., Tang L., Xue M., Zhuang Z., Yan Y., Valtchev V., Qiu S. (2020). Exfoliated Mesoporous 2D Covalent Organic Frameworks for High-Rate Electrochemical Double-Layer Capacitors. Adv. Mater..

[95] Evans A.M., Bradshaw N.P., Litchfield B., Strauss M.J., Seckman B., Ryder M.R., Castano I., Gilmore C., Gianneschi N.C., Mulzer C.R. (2020). High-Sensitivity Acoustic Molecular Sensors Based on Large-Area, Spray-Coated 2D Covalent Organic Frameworks. Adv. Mater..

[96] Chen W., Wang L., Mo D., He F., Wen Z., Wu X., Xu H., Chen L. (2020). Modulating Benzothiadiazole-Based Covalent Organic Frameworks via Halogenation for Enhanced Photocatalytic Water Splitting. Angew. Chem. Int. Ed. Engl..

[97] Gao P., Shen X., Liu X., Chen Y., Pan W., Li N., Tang B. (2021). Nucleic Acid-Gated Covalent Organic Frameworks for Cancer-Specific Imaging and Drug Release. Anal. Chem..

[98] Keller N., Bein T. (2021). Optoelectronic processes in covalent organic frameworks. Chem. Soc. Rev..

[99] Liu S., Qian T., Wang M., Ji H., Shen X., Wang C., Yan C. (2021). Proton-filtering covalent organic frameworks with superior nitrogen penetration flux promote ambient ammonia synthesis. Nat. Catal..

[100] Zhi Y., Wang Z., Zhang H.L., Zhang Q. (2020). Recent Progress in Metal-Free Covalent Organic Frameworks as Heterogeneous Catalysts. Small.

[101] Zheng Z.-J., Ye H., Guo Z.-P. (2021). Recent progress on pristine metal/covalent-organic frameworks and their composites for lithium–sulfur batteries. Energy Environ. Sci..

[102] Xu X., Wang S., Yue Y., Huang N. (2020). Semiconductive Porphyrin-Based Covalent Organic Frameworks for Sensitive Near-Infrared Detection. ACS Appl. Mater. Interfaces.

[103] Caponetti V., Trzcinski J.W., Cantelli A., Tavano R., Papini E., Mancin F., Montalti M. (2019). Self-Assembled Biocompatible Fluorescent Nanoparticles for Bioimaging. Front. Chem..

[104] Ding F., Zhan Y., Lu X., Sun Y. (2018). Recent advances in near-infrared II fluorophores for multifunctional biomedical imaging. Chem. Sci..

[105] Guest M., Mir R., Foran G., Hickson B., Necakov A., Dudding T. (2020). Trisaminocyclopropenium Cations as Small-Molecule Organic Fluorophores: Design Guidelines and Bioimaging Applications. J. Org. Chem..

[106] Guria S., Ghosh A., Upadhyay P., Das M.K., Mishra T., Adhikary A., Adhikari S. (2020). Small-Molecule Probe for Sensing Serum Albumin with Consequential Self-Assembly as a Fluorescent Organic Nanoparticle for Bioimaging and Drug-Delivery Applications. ACS Appl. Bio Mater..

[107] Lei Z., Sun C., Pei P., Wang S., Li D., Zhang X., Zhang F. (2019). Stable, Wavelength-Tunable Fluorescent Dyes in the NIR-II Region for In Vivo High-Contrast Bioimaging and Multiplexed Biosensing. Angew. Chem. Int. Ed. Engl..

[108] Li B., Lu L., Zhao M., Lei Z., Zhang F. (2018). An Efficient 1064 nm NIR-II Excitation Fluorescent Molecular Dye for Deep-Tissue High-Resolution Dynamic Bioimaging. Angew. Chem. Int. Ed. Engl..

[109] Li L., Sun H. (2020). Next Generation of Small-Molecule Fluorogenic Probes for Bioimaging. Biochemistry.

[110] Li Q., Ding Q., Li Y., Zeng X., Liu Y., Lu S., Zhou H., Wang X., Wu J., Meng X. (2020). Novel small-molecule fluorophores for in vivo NIR-IIa and NIR-IIb imaging. Chem. Commun..

[111] Li Y., Zhou H., Bi R., Li X., Zha M., Yang Y., Ni J.S., Liew W.H., Olivo M., Yao K. (2021). Acceptor engineering of small-molecule fluorophores for NIR-II fluorescence and photoacoustic imaging. J. Mater. Chem. B.

[112] Lin J., Zeng X., Xiao Y., Tang L., Nong J., Liu Y., Zhou H., Ding B., Xu F., Tong H. (2019). Novel near-infrared II aggregation-induced emission dots for in vivo bioimaging. Chem. Sci..

[113] Liu S., Chen R., Zhang J., Li Y., He M., Fan X., Zhang H., Lu X., Kwok R.T.K., Lin H. (2020). Incorporation of Planar Blocks into Twisted Skeletons: Boosting Brightness of Fluorophores for Bioimaging beyond 1500 Nanometer. ACS Nano.

[114] Wang S., Li B., Zhang F. (2020). Molecular Fluorophores for Deep-Tissue Bioimaging. ACS Cent. Sci..

[115] Yang M., Deng J., Su H., Gu S., Zhang J., Zhong A., Wu F. (2021). Small organic molecule-based nanoparticles with red/near-infrared aggregation-induced emission for bioimaging and PDT/PTT synergistic therapy. Mater. Chem. Front..

[116] Yang Z., Fan X., Li H., Li X., Li S., Zhang Z., Lin H., Qian J., Hua J. (2021). A Small-Molecule Diketopyrrolopyrrole-Based Dye for in vivo NIR-IIa Fluorescence Bioimaging. Chemistry.

[117] Alifu N., Zebibula A., Zhang H., Ni H., Zhu L., Xi W., Wang Y., Zhang X., Wu C., Qian J. (2020). NIR-IIb excitable bright polymer dots with deep-red emission for in vivo through-skull three-photon fluorescence bioimaging. Nano Res..

[118] Braeken Y., Cheruku S., Seneca S., Smisdom N., Berden L., Kruyfhooft L., Penxten H., Lutsen L., Fron E., Vanderzande D. (2019). Effect of Branching on the Optical Properties of Poly(p-phenylene ethynylene) Conjugated Polymer Nanoparticles for Bioimaging. ACS Biomater. Sci. Eng..

[119] Dineshkumar S., Raj A., Srivastava A., Mukherjee S., Pasha S.S., Kachwal V., Fageria L., Chowdhury R., Laskar I.R. (2019). Facile Incorporation of "Aggregation-Induced Emission"-Active Conjugated Polymer into Mesoporous Silica Hollow Nanospheres: Synthesis, Characterization, Photophysical Studies, and Application in Bioimaging. ACS Appl. Mater. Interfaces.

[120] Fang X., Ju B., Liu Z., Wang F., Xi G., Sun Z., Chen H., Sui C., Wang M., Wu C. (2019). Compact Conjugated Polymer Dots with Covalently Incorporated Metalloporphyrins for Hypoxia Bioimaging. ChembioChem.

[121] Koralli P.D., Nega A., Vagiaki L.E., Pavlou A., Siskos M.G., Dimitrakopoulou-Strauss A., Gregoriou V.G., Chochos C.L. (2020). New conjugated polymer nanoparticles with high photoluminescence quantum yields for far-red and near infrared fluorescence bioimaging. Mater. Chem. Front..

[122] Li H., Wu J., Yin J.F., Wang H., Wu Y., Kuang G.C. (2019). Photoresponsive, Water-Soluble Supramolecular Dendronized Polymer with Specific Lysosome-Targetable Bioimaging Application in Living Cells. Macromol. Rapid Commun..

[123] Yang J., Chen W., Chen X., Zhang X., Zhou H., Du H., Wang M., Ma Y., Jin X. (2021). Detection of Cu(2+) and S(2-) with fluorescent polymer nanoparticles and bioimaging in HeLa cells. Anal. Bioanal. Chem..

[124] Solhi E., Hasanzadeh M. (2019). Recent advances on the biosensing and bioimaging based on polymer dots as advanced nanomaterial: Analytical approaches. TrAC Trends Anal. Chem..

[125] Biswas M.C., Islam M.T., Nandy P.K., Hossain M.M. (2021). Graphene Quantum Dots (GQDs) for Bioimaging and Drug Delivery Applications: A Review. ACS Mater. Lett..

[126] Marković Z.M., Labudová M., Danko M., Matijašević D., Mičušík M., Nádaždy V., Kováčová M., Kleinová A., Špitalský Z., Pavlović V. (2020). Highly Efficient Antioxidant F- and Cl-Doped Carbon Quantum Dots for Bioimaging. ACS Sustain. Chem. Eng..

[127] Pandey S., Bodas D. (2020). High-quality quantum dots for multiplexed bioimaging: A critical review. Adv. Colloid Interface Sci..

[128] Atchudan R., Jebakumar Immanuel Edison T.N., Shanmugam M., Perumal S., Somanathan T., Lee Y.R. (2021). Sustainable synthesis of carbon quantum dots from banana peel waste using hydrothermal process for in vivo bioimaging. Phys. E Low-Dimens. Syst. Nanostructures.

[129] Chen W., Lv G., Hu W., Li D., Chen S., Dai Z. (2018). Synthesis and applications of graphene quantum dots: A review. Nanotechnol. Rev..

[130] Hu J., Sun Y., Aryee A.A., Qu L., Zhang K., Li Z. (2021). Mechanisms for carbon dots-based chemosensing, biosensing, and bioimaging: A review. Anal. Chim. Acta.

[131] Pandey P.K., Preeti, Rawat K., Prasad T., Bohidar H.B. (2020). Multifunctional, fluorescent DNA-derived carbon dots for biomedical applications: Bioimaging, luminescent DNA hydrogels, and dopamine detection. J. Mater. Chem. B.

[132] Meng Y., Jiao Y., Zhang Y., Li Y., Gao Y., Lu W., Liu Y., Shuang S., Dong C. (2020). Multi-sensing function integrated nitrogen-doped fluorescent carbon dots as the platform toward multi-mode detection and bioimaging. Talanta.

[133] Bogireddy N.K.R., Lara J., Fragoso L.R., Agarwal V. (2020). One-step hydrothermal preparation of highly stable N doped oxidized carbon dots for toxic organic pollutants sensing and bioimaging. Chem. Eng. J..

[134] Li H., Yan X., Kong D., Jin R., Sun C., Du D., Lin Y., Lu G. (2020). Recent advances in carbon dots for bioimaging applications. Nanoscale Horiz..

[135] Yue L., Li H., Sun Q., Zhang J., Luo X., Wu F., Zhu X. (2020). Red-Emissive Ruthenium-Containing Carbon Dots for Bioimaging and Photodynamic Cancer Therapy. ACS Appl. Nano Mater..

[136] Perumal S., Atchudan R., Edison T.N.J.I., Lee Y.R. (2021). Sustainable synthesis of multifunctional carbon dots using biomass and their applications: A mini-review. J. Environ. Chem. Eng..

[137] Bhatt M., Bhatt S., Vyas G., Raval I.H., Haldar S., Paul P. (2020). Water-Dispersible Fluorescent Carbon Dots as Bioimaging Agents and Probes for Hg2+ and Cu2+ Ions. ACS Appl. Nano Mater..

[138] Butler K.S., Pearce C.J., Nail E.A., Vincent G.A., Sava Gallis D.F. (2020). Antibody Targeted Metal-Organic Frameworks for Bioimaging Applications. ACS Appl. Mater. Interfaces.

[139] Guan Q.L., Han C., Bai F.Y., Liu J., Xing Y.H., Shi Z., Sun L.X. (2020). Bismuth-MOF based on tetraphenylethylene derivative as a luminescent sensor with turn-off/on for application of Fe3+ detection in serum and bioimaging, as well as emissive spectra analysis by TRES. Sens. Actuators B Chem..

[140] Zhang M., Gao Y., Han L., Zhu N., Gao X. (2020). The construction of a multifunctional metal–organic framework for targeting tumors and bioimaging. New J. Chem..

[141] Wang H.-S., Wang Y.-H., Ding Y. (2020). Development of biological metal–organic frameworks designed for biomedical applications: From bio-sensing/bio-imaging to disease treatment. Nanoscale Adv..

[142] Liu M., Ren X., Meng X., Li H. (2021). Metal-Organic Frameworks-Based Fluorescent Nanocomposites for Bioimaging in Living Cells and in vivo†. Chin. J. Chem..

[143] Song Y., Yang J., Wang L., Xie Z. (2020). Metal-Organic Sheets for Efficient Drug Delivery and Bioimaging. ChemMedChem.

[144] Sohrabi H., Javanbakht S., Oroojalian F., Rouhani F., Shaabani A., Majidi M.R., Hashemzaei M., Hanifehpour Y., Mokhtarzadeh A., Morsali A. (2021). Nanoscale Metal-Organic Frameworks: Recent developments in synthesis, modifications and bioimaging applications. Chemosphere.

[145] Fu D.-Y., Liu X., Zheng X., Zhou M., Wang W., Su G., Liu T., Wang L., Xie Z. (2022). Polymer-metal-organic framework hybrids for bioimaging and cancer therapy. Coord. Chem. Rev..

[146] Naskar K., Bhanja A.K., Paul S., Pal K., Sinha C. (2020). Trace Quantity Detection of H2PO4– by Fluorescent Metal–Organic Framework (F-MOF) and Bioimaging Study. Cryst. Growth Des..

[147] Feng K., Hao H., Huang F., Lang X., Wang C. (2021). A 2D porphyrin-based covalent organic framework with TEMPO for cooperative photocatalysis in selective aerobic oxidation of sulfides. Mater. Chem. Front..

[148] Zeng J.Y., Wang X.S., Xie B.R., Li M.J., Zhang X.Z. (2020). Covalent Organic Framework for Improving Near-Infrared Light Induced Fluorescence Imaging through Two-Photon Induction. Angew. Chem. Int. Ed. Engl..

[149] Gao P., Tang K., Lou R., Liu X., Wei R., Li N., Tang B. (2021). Covalent Organic Framework-Based Spherical Nucleic Acid Probe with a Bonding Defect-Amplified Modification Strategy. Anal. Chem..

[150] Gao P., Shen X., Liu X., Cui B., Wang M., Wan X., Li N., Tang B. (2021). Covalent Organic Framework-Derived Carbonous Nanoprobes for Cancer Cell Imaging. ACS Appl. Mater. Interfaces.

[151] Li B., Lv Y.-K., Wang Z.-D., Peng P., Zang S.-Q. (2021). Edge confined covalent organic framework with efficient biocompatibility and photothermic conversion. Nano Today.

[152] Ma J., Shu T., Sun Y., Zhou X., Ren C., Su L., Zhang X. (2022). Luminescent Covalent Organic Frameworks for Biosensing and Bioimaging Applications. Small.

[153] Gao P., Wei R., Chen Y., Liu X., Zhang J., Pan W., Li N., Tang B. (2021). Multicolor Covalent Organic Framework-DNA Nanoprobe for Fluorescence Imaging of Biomarkers with Different Locations in Living Cells. Anal. Chem..

[154] Cao Y., Zhang L., Yang J., Zhang J., Si W., Wang J., Iqbal A., Qin W., Liu Y. (2021). Ratiometric covalent organic framework florescence sensor for detecting hydrazine produced from isoniazid metabolism in cell. Sens. Actuators B Chem..

[155] He Z., Ke C., Tang B.Z. (2018). Journey of Aggregation-Induced Emission Research. ACS Omega.

[156] Wang J., Zhao Y., Dou C., Sun H., Xu P., Ye K., Zhang J., Jiang S., Li F., Wang Y. (2007). Alkyl and Dendron Substituted Quinacridones:  Synthesis, Structures, and Luminescent Properties. J. Phys. Chem. B.

[157] Hecht S., Fréchet J.M.J. (2001). Dendritic Encapsulation of Function: Applying Nature’s Site Isolation Principle from Biomimetics to Materials Science. Angew. Chem. Int. Ed..

[158] Nguyen B.T., Gautrot J.E., Ji C., Brunner P.-L., Nguyen M.T., Zhu X.X. (2006). Enhancing the Photoluminescence Intensity of Conjugated Polycationic Polymers by Using Quantum Dots as Antiaggregation Reagents. Langmuir.

[159] Taylor P.N., O’Connell M.J., McNeill L.A., Hall M.J., Aplin R.T., Anderson H.L. (2000). Insulated Molecular Wires: Synthesis of Conjugated Polyrotaxanes by Suzuki Coupling in Water. Angew. Chem. Int. Ed..

[160] Chen L., Xu S., McBranch D., Whitten D. (2000). Tuning the Properties of Conjugated Polyelectrolytes through Surfactant Complexation. J. Am. Chem. Soc..

[161] Hong Y., Lam J.W.Y., Tang B.Z. (2011). Aggregation-induced emission. Chem. Soc. Rev..

[162] Peng Z., Wang Z., Tong B., Ji Y., Shi J., Zhi J., Dong Y. (2016). Anthracene Modified by Aldehyde Groups Exhibiting Aggregation-Induced Emission Properties. Chin. J. Chem..

[163] Huang M., Yu R., Xu K., Ye S., Kuang S., Zhu X., Wan Y. (2016). An arch-bridge-type fluorophore for bridging the gap between aggregation-caused quenching (ACQ) and aggregation-induced emission (AIE). Chem. Sci..

[164] Shen X.Y., Wang Y.J., Zhang H., Qin A., Sun J.Z., Tang B.Z. (2014). Conjugates of tetraphenylethene and diketopyrrolopyrrole: Tuning the emission properties with phenyl bridges. Chem. Commun..

[165] Guo B., Cai X., Xu S., Fateminia S.M.A., Liu J., Liang J., Feng G., Wu W., Liu B. (2016). Decoration of porphyrin with tetraphenylethene: Converting a fluorophore with aggregation-caused quenching to aggregation-induced emission enhancement. J. Mater. Chem. B.

[166] Dong L., Shang G., Shi J., Zhi J., Tong B., Dong Y. (2017). Effect of Substituent Position on the Photophysical Properties of Triphenylpyrrole Isomers. J. Phys. Chem. C.

[167] Zong L., Xie Y., Wang C., Li J.-R., Li Q., Li Z. (2016). From ACQ to AIE: The suppression of the strong π–π interaction of naphthalene diimide derivatives through the adjustment of their flexible chains. Chem. Commun..

[168] Naito H., Morisaki Y., Chujo Y. (2015). o-Carborane-Based Anthracene: A Variety of Emission Behaviors. Angew. Chem. Int. Ed..

[169] Peng Z., Ji Y., Huang Z., Tong B., Shi J., Dong Y. (2018). A strategy for the molecular design of aggregation-induced emission units further modified by substituents. Mater. Chem. Front..

[170] Bu F., Duan R., Xie Y., Yi Y., Peng Q., Hu R., Qin A., Zhao Z., Tang B.Z. (2015). Unusual Aggregation-Induced Emission of a Coumarin Derivative as a Result of the Restriction of an Intramolecular Twisting Motion. Angew. Chem. Int. Ed..

[171] Belmonte-Vázquez J.L., Amador-Sánchez Y.A., Rodríguez-Cortés L.A., Rodríguez-Molina B. (2021). Dual-State Emission (DSE) in Organic Fluorophores: Design and Applications. Chem. Mater..

[172] Tu T.K.T., Salma S.A., Jeong M., Kim J.H., Jeong Y.T., Gal Y.-S., Lim K.T. (2021). Carbazole-Based Polyimide as a Hole-Transporting Material for Optoelectronic Applications. Macromol. Res..

[173] Jayabharathi J., Thilagavathy S., Thanikachalam V. (2021). Non-doped OLEDs based on tetraphenylethylene phenanthroimidazoles with negligible efficiency roll-off: Effects of end group regulated stimulus responsive AIE luminogens. Mater. Adv..

[174] Ye F., Chen W., Pan Y., Liu S.H., Yin J. (2019). Benzobisthiadiazoles: From structure to function. Dye. Pigment..

[175] Farnum D.G., Mehta G., Moore G.G.I., Siegal F.P. (1974). Attempted reformatskii reaction of benzonitrile, 1,4-diketo-3,6-diphenylpyrrolo[3,4-C]pyrrole. A lactam analogue of pentalene. Tetrahedron Lett..

[176] Tang A., Zhan C., Yao J., Zhou E. (2017). Design of Diketopyrrolopyrrole (DPP)-Based Small Molecules for Organic-Solar-Cell Applications. Adv. Mater..

[177] Abdelnasser S., Park G., Han H., Toth R., Yoon H. (2019). Enhanced photocatalytic performance of poly(3,4-ethylenedioxythiophene)-coated TiO2 nanotube electrodes. Synth. Met..

[178] Kwon O.S., Park S.J., Park H.-W., Kim T., Kang M., Jang J., Yoon H. (2012). Kinetically Controlled Formation of Multidimensional Poly(3,4-ethylenedioxythiophene) Nanostructures in Vapor-Deposition Polymerization. Chem. Mater..

[179] Park S.J., Park C.S., Yoon H. (2017). Chemo-Electrical Gas Sensors Based on Conducting Polymer Hybrids. Polymers.

[180] Kwon O.S., Park C.S., Park S.J., Noh S., Kim S., Kong H.J., Bae J., Lee C.-S., Yoon H. (2016). Carboxylic Acid-Functionalized Conducting-Polymer Nanotubes as Highly Sensitive Nerve-Agent Chemiresistors. Sci. Rep..

[181] Park S.J., Kwon O.S., Lee J.E., Jang J., Yoon H. (2014). Conducting Polymer-Based Nanohybrid Transducers: A Potential Route to High Sensitivity and Selectivity Sensors. Sensors.

[182] Yoon H. (2013). Current Trends in Sensors Based on Conducting Polymer Nanomaterials. Nanomaterials.

[183] Lee J.E., Park S.J., Kwon O.S., Shim H.W., Jang J., Yoon H. (2014). Systematic investigation on charge storage behaviour of multidimensional poly(3,4-ethylenedioxythiophene) nanostructures. RSC Adv..

[184] Le T.-H., Kim Y., Yoon H. (2017). Electrical and Electrochemical Properties of Conducting Polymers. Polymers.

[185] Choi H., Ahn K.-J., Lee Y., Noh S., Yoon H. (2015). Free-Standing, Multilayered Graphene/Polyaniline-Glue/Graphene Nanostructures for Flexible, Solid-State Electrochemical Capacitor Application. Adv. Mater. Interfaces.

[186] Kang M., Lee J.E., Shim H.W., Jeong M.S., Im W.B., Yoon H. (2014). Intrinsically conductive polymer binders for electrochemical capacitor application. RSC Adv..

[187] Choi H., Yoon H. (2015). Nanostructured Electrode Materials for Electrochemical Capacitor Applications. Nanomaterials.

[188] Nguyen D.N., Yoon H. (2016). Recent Advances in Nanostructured Conducting Polymers: From Synthesis to Practical Applications. Polymers.

[189] Ahn K.-J., Lee Y., Choi H., Kim M.-S., Im K., Noh S., Yoon H. (2015). Surfactant-Templated Synthesis of Polypyrrole Nanocages as Redox Mediators for Efficient Energy Storage. Sci. Rep..

[190] Yoon H., Choi M., Lee K.J., Jang J. (2008). Versatile strategies for fabricating polymer nanomaterials with controlled size and morphology. Macromol. Res..

[191] Kwon O.S., Park S.J., Yoon H., Jang J. (2012). Highly sensitive and selective chemiresistive sensors based on multidimensional polypyrrole nanotubes. Chem. Commun..

[192] Kim Y.U., Park S.H., Nhan N.T., Hoang M.H., Cho M.J., Choi D.H. (2021). Optimal Design of PEDOT:PSS Polymer-Based Silver Nanowire Electrodes for Realization of Flexible Polymer Solar Cells. Macromol. Res..

[193] Liu Z., Deng C., Su L., Wang D., Jiang Y., Tsuboi T., Zhang Q. (2021). Efficient Intramolecular Charge-Transfer Fluorophores Based on Substituted Triphenylphosphine Donors. Angew. Chem. Int. Ed..

[194] La D.D., Bhosale S.V., Jones L.A., Bhosale S.V. (2018). Tetraphenylethylene-Based AIE-Active Probes for Sensing Applications. ACS Appl. Mater. Interfaces.

[195] Mu C., Zhang Z., Hou Y., Liu H., Ma L., Li X., Ling S., He G., Zhang M. (2021). Tetraphenylethylene-Based Multicomponent Emissive Metallacages as Solid-State Fluorescent Materials. Angew. Chem. Int. Ed. Engl..

[196] Huang G., Xia Q., Huang W., Tian J., He Z., Li B.S., Tang B.Z. (2019). Multiple Anti-Counterfeiting Guarantees from a Simple Tetraphenylethylene Derivative—High-Contrasted and Multi-State Mechanochromism and Photochromism. Angew. Chem. Int. Ed. Engl..

[197] Guo Z., Li G., Wang H., Zhao J., Liu Y., Tan H., Li X., Stang P.J., Yan X. (2021). Drum-like Metallacages with Size-Dependent Fluorescence: Exploring the Photophysics of Tetraphenylethylene under Locked Conformations. J. Am. Chem. Soc..

[198] Sabuj M.A., Huda M.M., Sarap C.S., Rai N. (2021). Benzobisthiadiazole-based high-spin donor–acceptor conjugated polymers with localized spin distribution. Mater. Adv..

[199] Dutta T., Pal K., Koner A.L. (2020). Cellular metabolic activity marker via selective turn-ON detection of transporter protein using nitrobenzoxadiazole-based fluorescent reporter. Sci. Rep..

[200] Zhang C., Chen Z., Yang C., Liang E., Yi J., Yu G., Yang C. (2018). Effects of Different Unsaturated-Linker-Containing Donors on Electronic Properties of Benzobisthiadiazole-Based Copolymers. Macromol. Chem. Phys..

[201] Wang Y., Tan A.T.-R., Mori T., Michinobu T. (2018). Inversion of charge carrier polarity and boosting the mobility of organic semiconducting polymers based on benzobisthiadiazole derivatives by fluorination. J. Mater. Chem. C.

[202] Wang R., Cai Q., Zhu Y., Mi Z., Weng W., Liu Y., Wan J., Hu J., Wang C., Yang D. (2021). An n-Type Benzobisthiadiazole-Based Covalent Organic Framework with Narrowed Bandgap and Enhanced Electroactivity. Chem. Mater..

[203] Li Y., Rajasree S.S., Lee G.Y., Yu J., Tang J.H., Ni R., Li G., Houk K.N., Deria P., Stang P.J. (2021). Anthracene-Triphenylamine-Based Platinum(II) Metallacages as Synthetic Light-Harvesting Assembly. J. Am. Chem. Soc..

[204] Siddique S.A., Siddique M.B.A., Hussain R., Liu X., Mehboob M.Y., Irshad Z., Adnan M. (2020). Efficient tuning of triphenylamine-based donor materials for high-efficiency organic solar cells. Comput. Theor. Chem..

[205] Yang Y., Wang J., Li D., Yang J., Fang M., Li Z. (2021). Tunable Photoresponsive Behaviors Based on Triphenylamine Derivatives: The Pivotal Role of pi-Conjugated Structure and Corresponding Application. Adv. Mater..

[206] Zou X., Cui S., Li J., Wei X., Zheng M. (2021). Diketopyrrolopyrrole Based Organic Semiconductor Materials for Field-Effect Transistors. Front. Chem..

[207] Zhang Q., Wang Q., Xu X., Liu J., Lu X., Huang W., Fan Q. (2021). Diketopyrrolopyrrole derivatives-based NIR-II fluorophores for theranostics. Dye. Pigment..

[208] Luo N., Zhang G., Liu Z. (2021). Keep glowing and going: Recent progress in diketopyrrolopyrrole synthesis towards organic optoelectronic materials. Org. Chem. Front..

[209] Koo D.G., Lee D., Noh J., Lee Y.H., Jang S., Nam I., Shin T.J., Park J. (2021). Impact of Intermolecular Interactions Between a Diketopyrrolopyrrole-Based Conjugated Polymer and Bromobenzaldehyde on Field-Effect Transistors. Macromol. Res..

[210] Cheon H.J., An T.K., Kim Y.-H. (2022). Diketopyrrolopyrrole (DPP)-Based Polymers and Their Organic Field-Effect Transistor Applications: A Review. Macromol. Res..

[211] Gunasekara D.S.W., Niu X., Lqbal W., He Y., Liu H. (2022). Pyrrole Coating with In Situ Polymerization for Piezoresistive Sensor Development—A Review. Macromol. Res..

[212] Xu P.Y., Zheng X., Kankala R.K., Wang S.B., Chen A.Z. (2021). Advances in Indocyanine Green-Based Codelivery Nanoplatforms for Combinatorial Therapy. ACS Biomater. Sci. Eng..

[213] Cho S.S., Salinas R., Lee J.Y.K. (2019). Indocyanine-Green for Fluorescence-Guided Surgery of Brain Tumors: Evidence, Techniques, and Practical Experience. Front. Surg..

[214] Yeroslavsky G., Umezawa M., Okubo K., Nigoghossian K., Thi Kim Dung D., Miyata K., Kamimura M., Soga K. (2020). Stabilization of indocyanine green dye in polymeric micelles for NIR-II fluorescence imaging and cancer treatment. Biomater. Sci..

[215] Zhang J., Yu H. (2021). Reduced energy loss enabled by thiophene-based interlayers for high performance and stable perovskite solar cells. J. Mater. Chem. A.

[216] da Cruz R.M.D., Mendonca-Junior F.J.B., de Melo N.B., Scotti L., de Araujo R.S.A., de Almeida R.N., de Moura R.O. (2021). Thiophene-Based Compounds with Potential Anti-Inflammatory Activity. Pharmaceuticals.

[217] Xu S., Sun H., Addicoat M., Biswal B.P., He F., Park S., Paasch S., Zhang T., Sheng W., Brunner E. (2021). Thiophene-Bridged Donor-Acceptor sp(2) -Carbon-Linked 2D Conjugated Polymers as Photocathodes for Water Reduction. Adv. Mater..

[218] Park S.H., Ahn J.-S., Kwon N.Y., Diem C.H., Harit A.K., Woo H.Y., Cho M.J., Choi D.H. (2021). Effect of Fused Thiophene Bridges on the Efficiency of Non-Fullerene Polymer Solar Cells made with Conjugated Donor Copolymers Containing Alkyl Thiophene-3-Carboxylate. Macromol. Res..

[219] Hu B., Zhang W., Wu J., Pang Z., Zhao S., Lu Z., Huang Y. (2019). An easily available near-infrared absorbing non-fullerene photovoltaic electron acceptor with indeno[1,2-b]indole as the central core. Dye. Pigment..

[220] Ding H., Chu Y., Xu M., Zhang S., Ye H., Hu Y., Hua J. (2020). Effect of π-bridge groups based on indeno[1,2-b]thiophene D–A–π–A sensitizers on the performance of dye-sensitized solar cells and photocatalytic hydrogen evolution. J. Mater. Chem. C.

[221] Simon Marques P., Tintori F., Andres Castan J.M., Josse P., Dalinot C., Allain M., Welch G., Blanchard P., Cabanetos C. (2020). Indeno[1,2-b]thiophene End-capped Perylene Diimide: Should the 1,6-Regioisomers be systematically considered as a byproduct?. Sci. Rep..

[222] Wu B., Cao B., Taylor I.M., Woeppel K., Cui X.T. (2019). Facile Synthesis of a 3,4-Ethylene-Dioxythiophene (EDOT) Derivative for Ease of Bio-Functionalization of the Conducting Polymer PEDOT. Front. Chem..

[223] Yu Q., Ding F., Shen J., He X. (2021). A newly nitrobenzoxadiazole (NBD)-fused reversible fluorescence probe for pH monitoring and application in bioimaging. Talanta.

[224] Siciliano G., Di Paolo V., Rotili D., Migale R., Pedini F., Casella M., Camerini S., Dalzoppo D., Henderson R., Huijs T. (2022). The Nitrobenzoxadiazole Derivative NBDHEX Behaves as Plasmodium falciparum Gametocyte Selective Inhibitor with Malaria Parasite Transmission Blocking Activity. Pharmaceuticals.

[225] Wang Z.L., Li F.F., Quach R., Ferrarese A., Forgiarini A., Ferrari M., D’Amore C., Bova S., Orso G., Fusi F. (2022). Nitrobenzoxadiazole derivatives of the rat selective toxicant norbormide as fluorescent probes for live cell imaging. Bioorg. Med. Chem..

[226] Robinson S.G., Yan Y., Hendriks K.H., Sanford M.S., Sigman M.S. (2019). Developing a Predictive Solubility Model for Monomeric and Oligomeric Cyclopropenium-Based Flow Battery Catholytes. J. Am. Chem. Soc..

[227] Ranga P.K., Ahmad F., Singh G., Tyagi A., Vijaya Anand R. (2021). Recent advances in the organocatalytic applications of cyclopropene- and cyclopropenium-based small molecules. Org. Biomol. Chem..

[228] Xu J., Xian A., Li Z., Liu J., Zhang Z., Yan R., Gao L., Liu B., Zhao L., Guo K. (2021). A Strained Ion Pair Permits Carbon Dioxide Fixation at Atmospheric Pressure by C-H H-Bonding Organocatalysis. J. Org. Chem..

[229] Yu Y.-B., Zhang Q., Wu L.-Y., Zhou Y.-L., Wang B.-X., Chen B.-Y., Hong J.-M. (2021). Reaction mechanism of N-(4-hydroxyphenyl)ethanamide electrodegradation via phosphorus-graphene prepared from triphenylphosphine: Generation and destruction of the reactive species. Chem. Eng. J..

[230] Silva D.E.S., Becceneri A.B., Santiago J.V.B., Gomes Neto J.A., Ellena J., Cominetti M.R., Pereira J.C.M., Hannon M.J., Netto A.V.G. (2020). Silver(I) complexes of 3-methoxy-4-hydroxybenzaldehyde thiosemicarbazones and triphenylphosphine: Structural, cytotoxicity, and apoptotic studies. Dalton Trans..

[231] Bormio Nunes J.H., Simoni D.A., Braga L.E.O., Ruiz A.L.T.G., Ernesto de Carvalho J., Corbi P.P. (2019). Synthesis, characterization, crystal structure and in vitro antiproliferative assays of the 2-thiouracilato(triphenylphosphine)gold(I) complex. J. Mol. Struct..

[232] Park C.S., Ha T.H., Choi S.A., Nguyen D.N., Noh S., Kwon O.S., Lee C.S., Yoon H. (2017). A near-infrared "turn-on" fluorescent probe with a self-immolative linker for the in vivo quantitative detection and imaging of hydrogen sulfide. Biosens. Bioelectron..

[233] Lei Z., Zhang F. (2021). Molecular Engineering of NIR-II Fluorophores for Improved Biomedical Detection. Angew. Chem. Int. Ed..

[234] Tang F., Liu J.-Y., Wu C.-Y., Liang Y.-X., Lu Z.-L., Ding A.-X., Xu M.-D. (2021). Two-Photon Near-Infrared AIE Luminogens as Multifunctional Gene Carriers for Cancer Theranostics. ACS Appl. Mater. Interfaces.

[235] Neto B.A.D., Lapis A.A.M., da Silva Júnior E.N., Dupont J. (2013). 2,1,3-Benzothiadiazole and Derivatives: Synthesis, Properties, Reactions, and Applications in Light Technology of Small Molecules. Eur. J. Org. Chem..

[236] Min D.J., Jillella R., Park S., Kang S., Park S.Y., Park J. (2021). Synthesis and Electro-Optical Properties of a New Conjugated Polymer Based on a Tetrazine Moiety for Solution-Processed Devices. Macromol. Res..

[237] Luo S., Zhang Q., Zhang Y., Weaver K.P., Phillip W.A., Guo R. (2018). Facile Synthesis of a Pentiptycene-Based Highly Microporous Organic Polymer for Gas Storage and Water Treatment. ACS Appl. Mater. Interfaces.

[238] Chu X.M., Wang C., Wang W.L., Liang L.L., Liu W., Gong K.K., Sun K.L. (2019). Triazole derivatives and their antiplasmodial and antimalarial activities. Eur. J. Med. Chem..

[239] Chen X., Dai W., Wu X., Su H., Chao C., Lei Y., Shi J., Tong B., Cai Z., Dong Y. (2021). Fluorene-based host-guest phosphorescence materials for information encryption. Chem. Eng. J..

[240] Sicard L., Quinton C., Lucas F., Jeannin O., Rault-Berthelot J., Poriel C. (2019). 1-Carbazolyl Spirobifluorene: Synthesis, Structural, Electrochemical, and Photophysical Properties. J. Phys. Chem. C.

[241] Arias-Coronado V.C., Pereira-Cameselle R., Ozcelik A., Talavera M., Pena-Gallego A., Alonso-Gomez J.L., Bolano S. (2019). Spirobifluorene Metallaaromatics. Chemistry.

[242] Guo H., Zhang H., Shen C., Zhang D., Liu S., Wu Y., Zhu W.H. (2021). A Coplanar pi-Extended Quinoxaline Based Hole-Transporting Material Enabling over 21 % Efficiency for Dopant-Free Perovskite Solar Cells. Angew. Chem. Int. Ed. Engl..

[243] Tariq S., Somakala K., Amir M. (2018). Quinoxaline: An insight into the recent pharmacological advances. Eur. J. Med. Chem..

[244] Chen D., Zhong Z., Ma Q., Shao J., Huang W., Dong X. (2020). Aza-BODIPY-Based Nanomedicines in Cancer Phototheranostics. ACS Appl. Mater. Interfaces.

[245] Shi Z., Han X., Hu W., Bai H., Peng B., Ji L., Fan Q., Li L., Huang W. (2020). Bioapplications of small molecule Aza-BODIPY: From rational structural design to in vivo investigations. Chem. Soc. Rev..

[246] Poddar M., Misra R. (2020). Recent advances of BODIPY based derivatives for optoelectronic applications. Coord. Chem. Rev..

[247] Chen J., Zhu Y., Kaskel S. (2021). Porphyrin-Based Metal-Organic Frameworks for Biomedical Applications. Angew. Chem. Int. Ed. Engl..

[248] Zhang X., Wasson M.C., Shayan M., Berdichevsky E.K., Ricardo-Noordberg J., Singh Z., Papazyan E.K., Castro A.J., Marino P., Ajoyan Z. (2021). A historical perspective on porphyrin-based metal–organic frameworks and their applications. Coord. Chem. Rev..

[249] Zhang P., O’Connor D., Wang Y., Jiang L., Xia T., Wang L., Tsang D.C.W., Ok Y.S., Hou D. (2020). A green biochar/iron oxide composite for methylene blue removal. J. Hazard. Mater..

[250] Alver E., Metin A.U., Brouers F. (2020). Methylene blue adsorption on magnetic alginate/rice husk bio-composite. Int. J. Biol. Macromol..

[251] Wang S., Liu J., Feng G., Ng L.G., Liu B. (2019). NIR-II Excitable Conjugated Polymer Dots with Bright NIR-I Emission for Deep In Vivo Two-Photon Brain Imaging Through Intact Skull. Adv. Funct. Mater..

[252] Alizadeh N., Ghasemi F., Salimi A., Hallaj R., Fathi F., Soleimani F. (2020). Polymer nanocomposite film for dual colorimetric and fluorescent ascorbic acid detection integrated single-cell bioimaging with droplet microfluidic platform. Dye. Pigment..

[253] Wang N., Ao H., Xiao W., Chen W., Li G., Wu J., Ju H. (2022). Confined electrochemiluminescence imaging microarray for high-throughput biosensing of single cell-released dopamine. Biosens. Bioelectron..

[254] Hu H., Yang C., Li M., Shao D., Mao H.Q., Leong K.W. (2021). Flash Technology-Based Self-Assembly in Nanoformulation: From Fabrication to Biomedical Applications. Mater. Today.

[255] Liu X., Wu W., Cui D., Chen X., Li W. (2021). Functional Micro-/Nanomaterials for Multiplexed Biodetection. Adv. Mater..

[256] Wang Y., Wang C., Li K., Song X., Yan X., Yu L., He Z. (2021). Recent advances of nanomedicine-based strategies in diabetes and complications management: Diagnostics, monitoring, and therapeutics. J. Control. Release.

[257] Wei J., Liu Y., Yu J., Chen L., Luo M., Yang L., Li P., Li S., Zhang X.H. (2021). Conjugated Polymers: Optical Toolbox for Bioimaging and Cancer Therapy. Small.

[258] Liu Y., Song Y., Zhang J., Yang Z., Peng X., Yan W., Qu J. (2021). Responsive Carbonized Polymer Dots for Optical Super-resolution and Fluorescence Lifetime Imaging of Nucleic Acids in Living Cells. ACS Appl. Mater. Interfaces.

[259] Zhou X., Liu Q., Yuan W., Li Z., Xu Y., Feng W., Xu C., Li F. (2021). Ultrabright NIR-II Emissive Polymer Dots for Metastatic Ovarian Cancer Detection. Adv. Sci..

